# A *Salmonella*-specific protein enhances the antimicrobial peptide response of the PhoPQ two-component system

**DOI:** 10.1128/mbio.01070-26

**Published:** 2026-10-09

**Authors:** Paris I. Brown, Luke A. F. Barretto, Casey C. Fowler

**Affiliations:** 1 Department of Biological Sciences, University of Alberta 3158, Edmonton, Alberta, Canada; NYU Langone Health, New York, New York, USA

**Keywords:** bacterial pathogenesis, pathogen evolution, *Salmonella enterica*, two-component regulatory systems, PhoP/PhoQ, antimicrobial peptides

## Abstract

*Salmonella enterica*, a major global pathogen, has evolved a distinctive pathogenesis that differs from related enteric pathogens. A central regulator of *Salmonella*-specific virulence gene expression is the broadly conserved PhoP/PhoQ (PhoPQ) two-component system. PhoPQ serves a distinct role in *Salmonella* compared to other species, suggesting a need to adapt its function for its unique and expanded role in this species. In this study, we identify a novel, *Salmonella*-specific component of the PhoPQ signaling system, a small putative lipoprotein we name PalA. We show that PalA localizes to the cytoplasmic membrane, where it interacts with PhoQ, leading to PhoP activation and a corresponding reprogramming of the PhoP regulon. PalA’s transcriptomic impact is entirely PhoPQ-dependent, indicating strict specificity. Upon cationic antimicrobial peptide exposure, *palA* expression is induced, and it strongly activates PhoPQ, but *palA* shows low expression and has a minimal effect under low pH or low Mg^2+^ conditions, indicating its impact on PhoPQ is signal-specific. Notably, PalA does not stimulate PhoPQ systems from closely related species, indicating that *Salmonella* PhoPQ has adapted to promote its activation by PalA. Collectively, this study unveils an evolutionary adaptation by *Salmonella* that enhances its virulence gene expression in response to cationic antimicrobial peptide exposure.

**IMPORTANCE:**

*Salmonella*, a major human pathogen, controls the expression of its virulence genes in part through the PhoP/PhoQ (PhoPQ) two-component system. In this study, we identify and characterize a novel component of this system: a small protein unique to *Salmonella*, which we name PalA. We show that exposure to cationic antimicrobial peptides (CAMPs), a host-derived signal encountered during infection, induces PalA expression. PalA then binds PhoQ, activating it and thereby enhancing virulence gene expression. This study reveals a previously overlooked component of the *Salmonella* PhoPQ signaling network, which is among the best-studied bacterial regulatory systems and has served as an important model system for understanding the regulation of bacterial pathogenesis. Furthermore, this work illustrates the important role small “adaptor proteins” can play in conferring new signaling properties to ancestral regulatory systems, in this case by boosting activity and the response to a specific environmental cue.

## INTRODUCTION

The Gram-negative bacterial pathogen *Salmonella enterica* is among the most burdensome foodborne pathogens, causing substantial morbidity and mortality throughout the world (1). Many *S. enterica* serovars have a broad host range and most commonly cause self-limited gastroenteritis in humans, whereas human-adapted serovars, such as serovar Typhi (*S*. Typhi), cause typhoid fever, a life-threatening systemic infection. *S. enterica* has a unique pathogenesis that stems from large numbers of horizontally acquired virulence factors, many of which are encoded on *Salmonella* Pathogenicity Islands (SPIs) (2, 3). *Salmonella* is a facultative intracellular pathogen, and it invades host cells to establish a unique replicative niche known as a *Salmonella*-containing vacuole (SCV) (4, 5). This is achieved largely through the actions of two Type III Secretion Systems (T3SSs) and their associated effector proteins: the SPI-1 encoded T3SS drives the initial cellular invasion, whereas the SPI-2 encoded T3SS mediates the maturation of SCV, among other functions (6, 7). *Salmonella* has evolved numerous mechanisms to support its SCV lifestyle, including factors to adapt to SCV environmental conditions and factors involved in host interactions (8, 9). For example, *S*. Typhi produces and secretes an A-B type toxin known as typhoid toxin from within SCVs that appears to be important for *S*. Typhi’s ability to cause long-term infections (a “carrier state”), which is essential for *S*. Typhi transmission (10–12).

The PhoP/PhoQ (PhoPQ) two-component system is a master regulator that controls the expression of many *Salmonella*-specific genes within the SCV (13). PhoQ is a sensor kinase that senses signals and responds by phosphorylating, and thereby activating, its cognate response regulator, PhoP, which is a DNA-binding transcription factor. The best-studied activating signals for *Salmonella* PhoQ are low magnesium conditions, mildly acidic pH, and cationic antimicrobial peptides (CAMPs) (13–17), all of which are cues that are present within SCVs (17–21). Other environmental signals have also been reported to modulate PhoPQ activity (13, 22, 23). Additional protein factors beyond the two core constituents also directly factor into the function of PhoPQ. Proteins that interact with and modify the activity of a TCS, herein referred to as adaptor proteins, are important for mediating PhoPQ’s output. MgrB, a widely conserved 47 amino acid membrane protein, binds PhoQ and suppresses its kinase activity, thereby reducing PhoP activity (24, 25). Furthermore, *Escherichia coli* and *S. enterica* each encode an additional adaptor protein that, in contrast to MgrB, stimulates PhoPQ activity. Interestingly, the *E. coli* protein (SafA) and the *S. enterica* protein (UgtL) are both specifically produced under low pH conditions, enhancing PhoPQ activity when this cue is present (26–28). PhoP directly or indirectly regulates large numbers of genes. When PhoPQ activity is stimulated within SCVs, this leads to the repression of genes involved in invasion (motility and SPI-1 T3SS) and the induction of a large regulon that includes the SPI-2 T3SS, the typhoid toxin genes, and genes that modify the cell envelope to adapt to harsh SCV conditions, amongst others (13, 29–33). PhoPQ’s central role within SCVs is well established, but its importance outside of the SCV is less clear. However, *Salmonella* presumably encounters PhoPQ-activating cues at extracellular stages of infection, such as low pH as *Salmonella* transits from the stomach through the intestines, or CAMPs secreted by the host and/or the resident microbiota within the lumen of the intestinal tract, and thus PhoPQ may serve relevant roles extracellularly as well (34, 35).

PhoPQ’s role in regulating large numbers of *Salmonella*-specific genes within a *Salmonella*-specific SCV niche is interesting, since it is not a *Salmonella*-specific TCS. PhoPQ is widely distributed throughout Gammaproteobacteria, where it appears to have a conserved role in magnesium homeostasis, in addition to serving other regulatory roles that can vary between species (13). It stands to reason that the function of PhoPQ would have needed to evolve in order for it to optimally fulfill its unique and expanded role in *Salmonella*. Indeed, several studies support this notion. For example, certain *Salmonella* PhoP promoter architectures appear to differ compared to those found in other species (36, 37), and *Salmonella* PhoPQ exhibits increased activity in response to its various activating cues compared to its close orthologs (38). In this study, we provide new insight into this issue by identifying a novel *Salmonella*-specific PhoPQ adaptor protein that enhances the activity of this TCS in a signal-specific manner, thereby enhancing the expression of PhoPQ’s virulence regulon when certain host-derived cues are encountered.

## RESULTS

### Identification of PalA, a putative lipoprotein that activates PhoPQ

**Fig 1:**
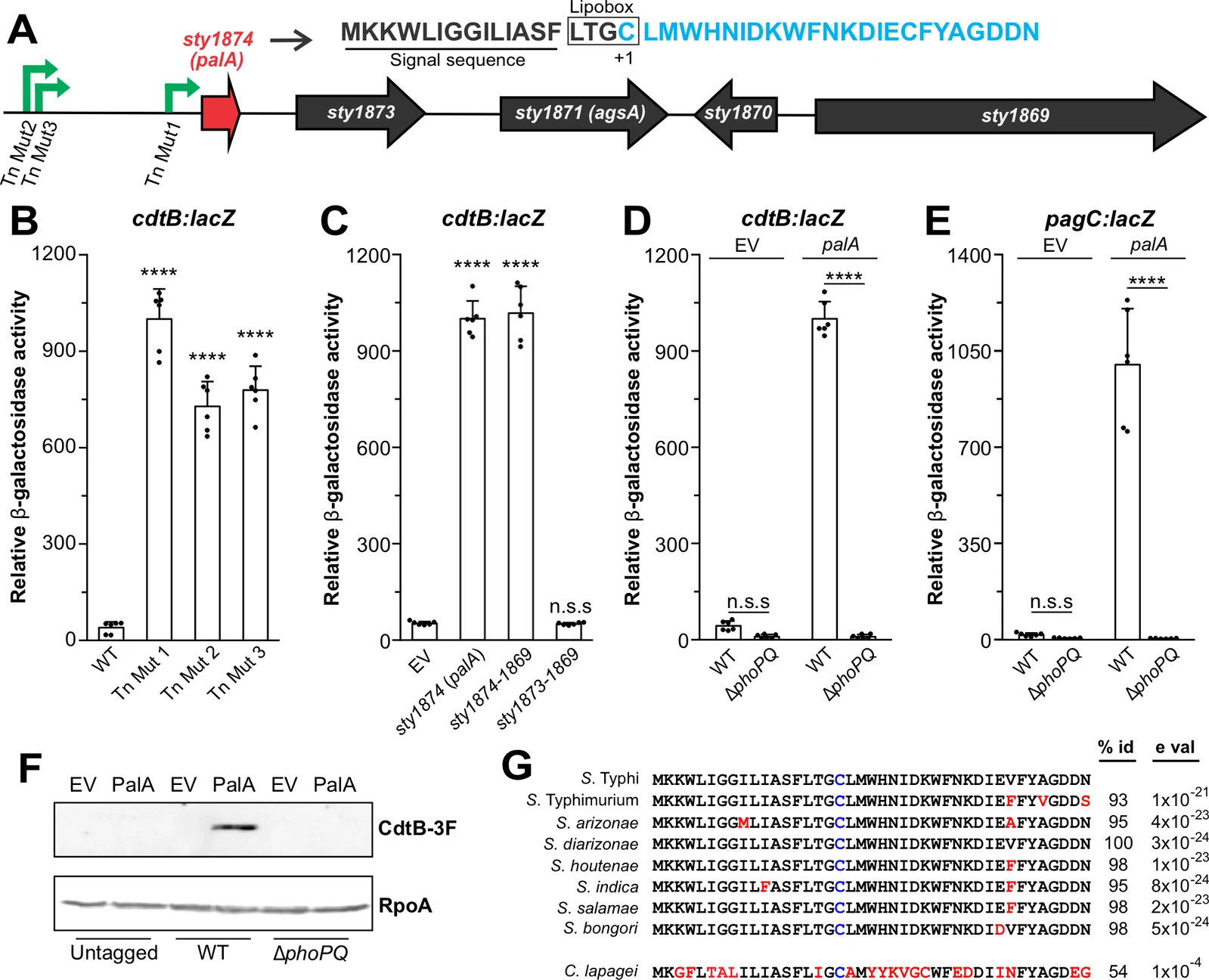
*sty1874* (*palA*) encodes a *Salmonella*-specific lipoprotein that induces the expression of PhoP-regulated genes. (**A**) Depiction of the *sty1874* genomic locus. Green bent arrows show the insertion sites of transposon (Tn) mutants that induce *cdtB:lacZ* activity; arrows show the direction of the outward-facing promoter responsible for *cdtB:lacZ* induction. The complete amino acid sequence of PalA is shown, where blue residues represent the predicted sequence of the processed PalA lipoprotein. (**B**) β-galactosidase activity in WT or transposon mutant *cdtB:lacZ S*. Typhi reporter strains following 6 h of growth in LB + 1 mM IPTG. (**C**) β-galactosidase activity in *S*. Typhi *cdtB:lacZ* carrying pBAD18-based plasmids encoding the *sty1874* gene alone, *sty1869-1874*, *sty1869-1873*, or an empty vector (EV) following 6 h growth in LB + 0.2% arabinose. (**D and E**) β-galactosidase activity in WT or Δ*phoPQ S*. Typhi *cdtB:lacZ* (**D**) or *pagC:lacZ* (**E**) carrying pBAD18-based plasmids encoding EV or *sty1874* (*palA*) following 6 h growth in LB + 0.2% arabinose. Bars represent the average of six biological replicates from two independent experiments (*n* = 6), and the error bars represent standard deviation. Statistical differences between the WT (B) or EV (C) and the indicated sample were calculated using one-way ANOVA using a Tukey’s post hoc test and are indicated as follows: n.s.s, not statistically significant; *P < 0.05, **P < 0.01, ***P < 0.001, ****P < 0.0001. For panels D and E, statistical differences between indicated samples were calculated using two-tailed *t*-tests. (**F**) Western blot analysis of CdtB-3×FLAG (CdtB-3F) levels from WT and Δ*phoPQ S*. Typhi *cdtB-3F* strains, as well as an untagged control strain carrying EV or palA-expressing plasmids following 6 h growth in LB + 0.2% arabinose. The α-RpoA antibody was used as a loading control. This experiment was repeated three times with equivalent results. (**G**) Alignment of PalA sequence from representative strains of diverse *Salmonella* lineages, as well as the closest PalA homolog identified outside of the *Salmonella* genus, *Cedecea lapagei* strain NCTC11466. Red residues show amino acid differences relative to *S*. Typhi PalA. Percent identity (% id) and E-values were obtained from NCBI BLASTp searches using *S*. Typhi PalA as the query sequence.

We previously used transposon mutagenesis to identify mutants that express the *S*. Typhi typhoid toxin genes under noninducing conditions, which identified H–NS as a transcriptional silencer of toxin expression (31). Although they were not investigated in the previous study, this screen also identified transposon insertions that induced expression of *cdtB:lacZ* (a genomically encoded transcriptional reporter for a typhoid toxin subunit) wherein the transposon’s outward-facing promoter inserted shortly upstream of the *sty1874* gene (Fig. 1A and B). This suggested that expressing a gene (or genes) at the *sty1874* genomic locus induces *cdtB* expression under noninducing growth conditions. To investigate this, we overexpressed *sty1874*, the full gene cluster at the *sty1874* locus (*sty1874-1869*), or the full gene cluster except for *sty1874* (*sty1873-186*9) in the *cdtB:lacZ* reporter strain and assayed for β-galactosidase activity. Overexpression of *sty1874* was observed to be both required and sufficient for *cdtB:lacZ* induction (Fig. 1C). On the basis of findings described below, we have named *sty1874* “*palA*” (PhoPQ-activating lipoprotein A).

*cdtB* is directly regulated by PhoPQ; PhoP activity is both necessary and sufficient for *cdtB* expression (31). We therefore hypothesized that PalA might operate via PhoPQ. Consistent with this, we found that *palA* expression failed to induce *cdtB:lacZ* in a Δ*phoP*/Δ*phoQ* (Δ*phoPQ*) strain (Fig. 1D). To further explore the possibility that PalA might activate PhoPQ, we tested the effect of overexpressing PalA on the expression of another well-characterized PhoP-activated gene, *pagC*, and found that its expression was also strongly induced by PalA in a PhoPQ-dependent fashion (Fig. 1E). Similar to our reporter assay results, we determined that CdtB protein levels were also markedly increased by *palA* overexpression, but not in the Δ*phoPQ* strain (Fig. 1F). Collectively, these data suggested that PalA expression results in increased PhoP activity and thus increased expression of PhoP-induced genes.

*palA* encodes a protein of unknown function that has a canonical Gram-negative lipobox signal sequence; it is predicted with very high confidence to be a lipoprotein by prediction programs (e.g., SignalP v5.0 [39], probability > 99.8%). It is very small, with a predicted size of only 23 amino acids in its mature form (Fig. 1A). Although it is not annotated in most *Salmonella* genomes, bioinformatic analysis revealed that PalA is well-conserved throughout the *Salmonella* genus, including *S. bongori* and all subspecies of *S. enterica* (Fig. 1G). *palA* is found within the SPI-11 region of the genome, and tBLASTn and BLASTp analyses revealed few significant hits for PalA outside *Salmonella*, the most significant of which were from *Cedecea* spp. However, this putative protein shares only ~54% amino acid sequence identity with PalA, much of which is in the signal sequence (Fig. 1G). Together, this indicates that PalA is a *Salmonella*-specific putative lipoprotein that is well conserved within this genus.

### PalA enhances PhoPQ activation in a signal-dependent manner

**Fig 2:**
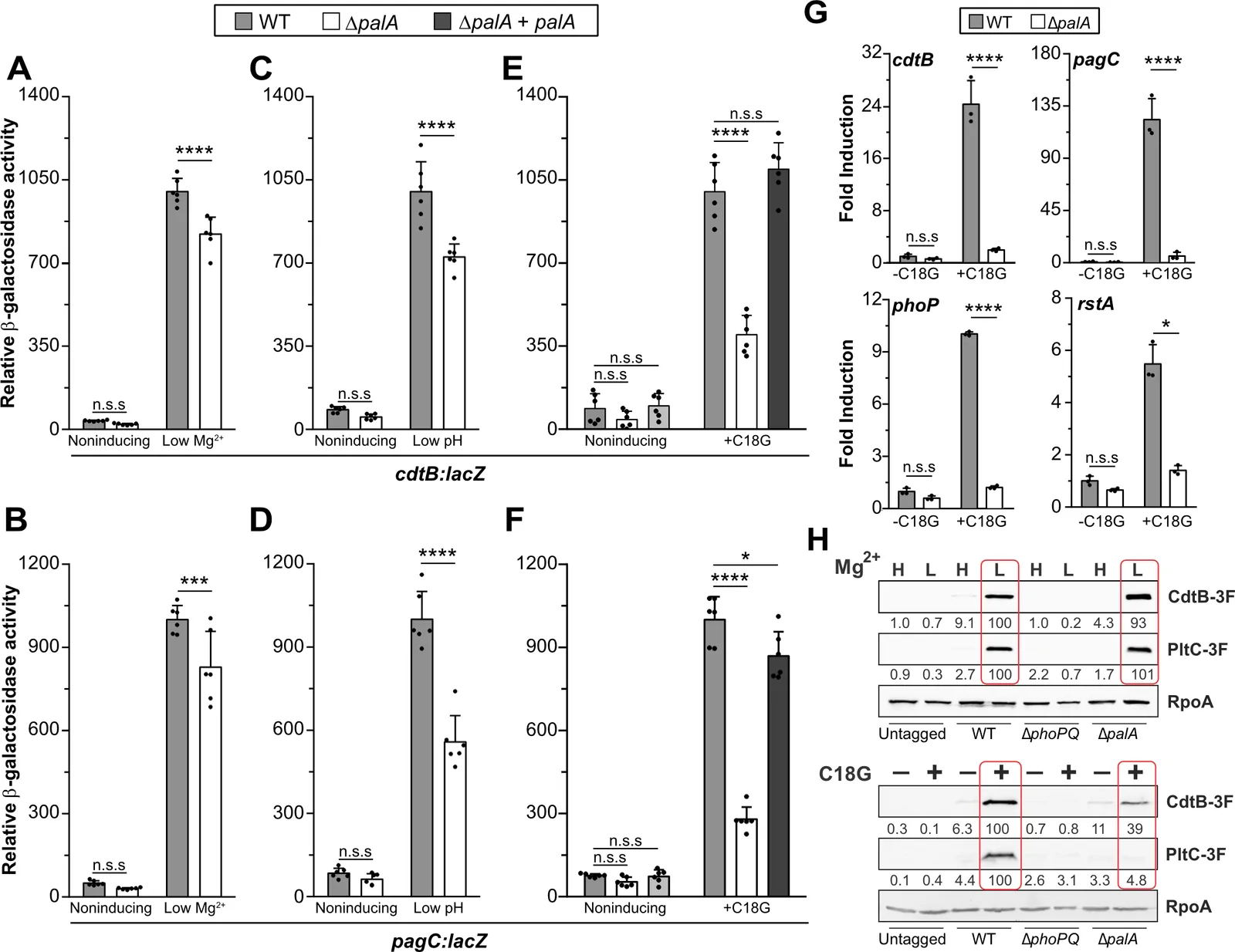
PalA enhances activation of the PhoPQ two-component system in the presence of cationic antimicrobial peptides. (**A–F**) β-galactosidase assays comparing WT and Δ*palA cdtB:lacZ* or *pagC:lacZ* reporter strains. Strains were grown for 6 h in noninducing TTIM, or TTIM with low Mg^2+^ (10 µM), low pH (4.9), or in the presence of 5 µg/mL C18G, as indicated. Panels E and F include a Δ*palA* strain where *palA* was restored to its genomic locus (Δ*palA + palA*). Data points represent values from six biological replicates over two independent experiments (*n* = 6). (**G**) WT or Δ*palA S*. Typhi was grown in TTIM 2 mM Mg^2+^ + 5 µg/mL C18G for 3 h. RNA was extracted, and RT-qPCR was used to quantify transcript levels of the indicated genes normalized to the control gene *rpoB*, and fold induction over the average WT noninducing value was calculated. Data points represent three biological replicates for each sample, and bars represent the average value; data are representative of two independent experiments with similar results. For panels A–G, error bars represent standard deviation, and statistical differences between the indicated samples were calculated using two-tailed *t*-tests (A–D, G) or one-way ANOVA using a Tukey’s post hoc test (E and F): n.s.s, not statistically significant; *P < 0.05, **P < 0.01, ***P < 0.001, ****P < 0.0001. (**H**) The indicated *cdtB-3F*/*pltC-3F* strains and an untagged control strain were grown for 6 h in the indicated conditions, and western blots were conducted on lysates using an α-FLAG antibody and α-RpoA antibody (loading control). “H” and “−” indicate noninducing conditions (TTIM 2 mM Mg^2+^ pH 7.2), “L” indicates TTIM with low (10 µM) Mg^2+^, and “+” indicates TTIM with 5 µg/mL C18G. Values below each band represent its average quantified value from three experiments.

Since PalA expression stimulates PhoP activity, we reasoned that it might play a role in PhoPQ-mediated gene regulation. We therefore compared WT and Δ*palA* strains for *cdtB:lacZ* and *pagC:lacZ* reporter activity in variations of the defined growth medium TTIM, including the base version, which lacks PhoPQ-inducing signals (pH 7.2, 2 mM Mg^2+^—“noninducing”), as well as TTIM with low Mg^2+^ (10 μM), an acidic pH (4.9), or supplemented with a cationic antimicrobial peptide (C18G) (38). We found that, while reporter activity in WT and Δ*palA* strains did not significantly differ under noninducing conditions, there was a small but statistically significant reduction in both *cdtB:lacZ* and *pagC:lacZ* expression in low Mg^2+^ and low pH conditions in the Δ*palA* strains (Fig. 2A through D). Importantly, however, when grown in the presence of C18G, the Δ*palA* strains showed a much more pronounced reduction in reporter activity (Fig. 2E and F). Restoring *palA* to its native locus in the Δ*palA* strain rescued activity, confirming that this CAMP signaling defect was due to the absence of *palA* and not a second-site mutation (Fig. 2E and F). To independently confirm PalA’s activity when exposed to CAMPs, we used RT-qPCR to quantify mRNA levels of four well-characterized PhoP-regulated genes—*cdtB*, *pagC*, *rstA*, and *phoP*—and observed that, compared to WT, the Δ*palA* strain had significantly reduced transcript levels for all of these genes in the TTIM+C18G growth condition (Fig. 2G). By contrast, a similar experiment comparing transcript levels in WT and Δ*palA* strains using low pH induction showed that *palA* had little or no effect on the expression of the same set of genes in this growth condition (Fig. S1). To further evaluate PalA’s contribution to PhoPQ signaling, we generated WT and Δ*palA* strains encoding 3×FLAG (3F)-tagged versions of both CdtB and PltC. PltC, a typhoid toxin delivery subunit, is indirectly regulated by PhoPQ via PhoPQ’s regulation of the SsrA/SsrB TCS, which in turn modulates *pltC* expression (40). We observed that in low Mg^2+^ TTIM, CdtB-3F and PltC-3F levels did not differ between the WT and Δ*palA* strains, whereas in TTIM+C18G, CdtB-3F and PltC-3F levels were reduced by >60% and >95%, respectively, in the Δ*palA* strain compared to WT (Fig. 2H). PalA’s substantial impact on PltC levels indicates that its activity reverberates beyond PhoPQ’s direct regulon. Collectively, these data indicate that PalA is an important constituent of the PhoPQ signaling program, and that its impact on PhoPQ activity is much more pronounced when PhoPQ is stimulated by CAMPs than by low pH or low Mg^2+^ conditions.

### *palA* expression is PhoPQ-dependent and induced by CAMPs

**Fig 3:**
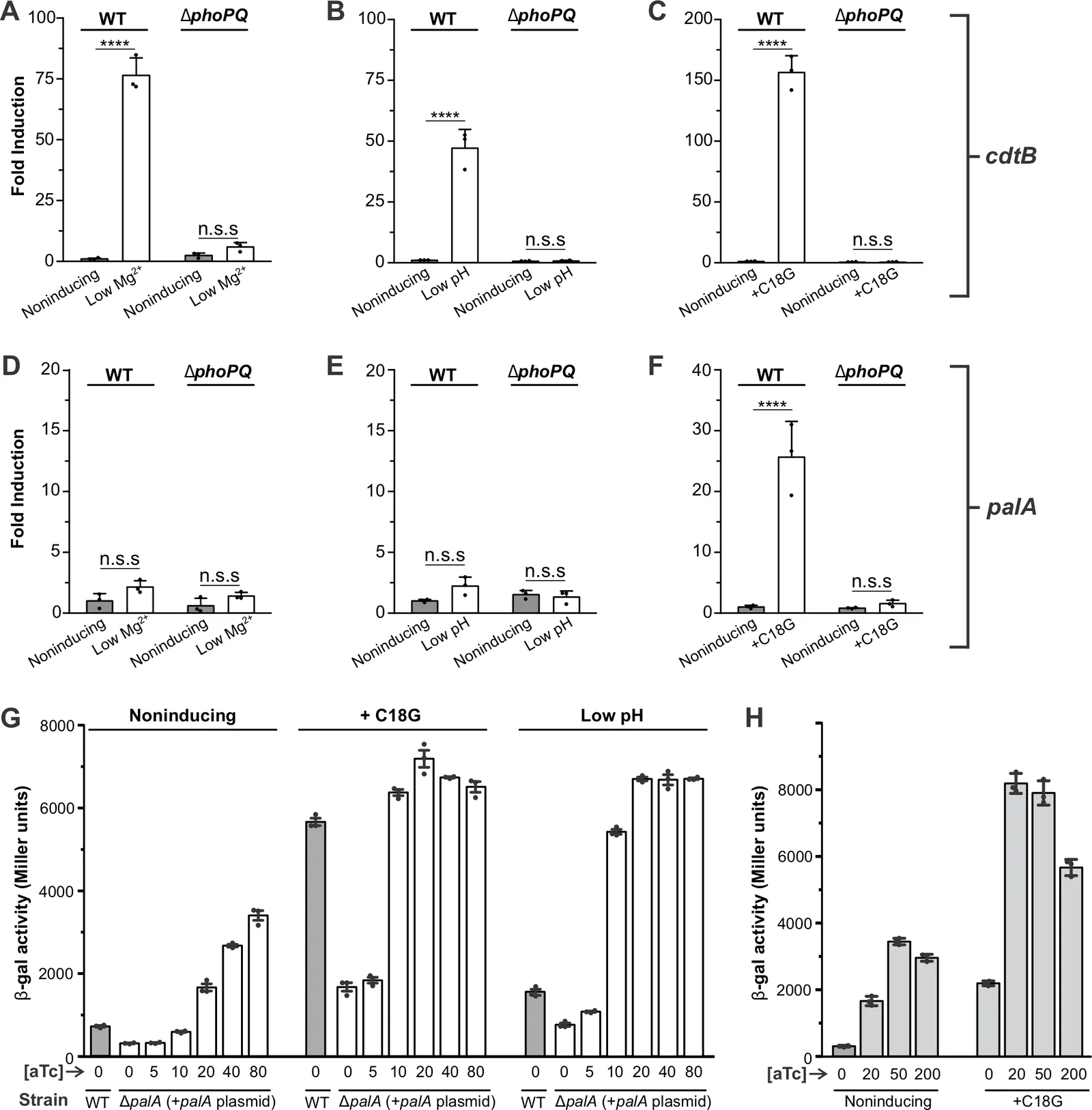
*palA* is induced by the CAMP C18G in a PhoPQ-dependent manner. (**A–F**) WT and ∆*phoPQ S*. Typhi were grown in noninducing TTIM (2 mM Mg^2+^, pH 7.2) or in TTIM with the indicated PhoPQ inducing conditions for either 6 h (low Mg^2+^), 4 h (low pH), or 2 h (+C18G). RNA was isolated, and transcript levels were analyzed using RT-qPCR. Transcript levels were normalized to *rpoB*. Data points represent the fold increase in RNA levels for *cdtB* (A–C) or *palA* (D–F) in the specified strain and inducing condition relative to the average value of the noninducing WT samples from that experiment. (**G**) β-galactosidase assays comparing a WT *pagC:lacZ* reporter strain to a similar Δ*palA* strain carrying a low-copy plasmid where *palA* is expressed from the P_tet_ promoter in response to the aTc inducer. Strains were grown for 4 h in noninducing TTIM, TTIM with a low pH (4.9), or TTIM containing 5 µg/mL C18G, containing the indicated concentration of aTc (in ng/mL). (**H**) The same *pagC:lacZ ΔpalA* + palA plasmid from panel G was similarly assayed using an expanded range of aTc concentrations under noninducing and C18G conditions. Bars represent average values for three biological replicates, and error bars represent standard deviation. Statistical differences between indicated samples were calculated using one-way ANOVA with Tukey’s post hoc test and are represented as follows: n.s.s, not statistically significant; ****P < 0.0001.

We next sought to explore the cause of PalA’s signal-specific effects. *palA* expression activates the PhoPQ system under otherwise noninducing conditions (Fig. 1), indicating that it does not require specific conditions (e.g., CAMP) to stimulate PhoPQ, at least not when it is overexpressed. We therefore speculated that PalA’s signal-dependent effects might stem from its genetic regulation. To test this, we quantified *palA* transcript levels under various PhoP-activating conditions in WT or ∆*phoPQ* strains. As expected, transcript levels of our positive control gene *cdtB* were induced by low Mg^2+^, low pH, and C18G in a PhoPQ-dependent manner (Fig. 3A through C). For *palA*, we found that growth in TTIM+C18G caused a substantial induction (~20×), but that low Mg^2+^ and low pH conditions yielded modest increases in *palA* transcript level that did not reach statistical significance (Fig. 3D through F). Importantly, we found that *palA* transcription in TTIM+C18G is dependent upon PhoPQ, suggesting that PalA forms a positive feedback loop wherein the presence of CAMPs induces PhoP upregulation of *palA*, which in turn amplifies the PhoPQ response. The observation that PhoP-inducing conditions, such as low Mg^2+^, do not strongly induce *palA* expression indicates that, in addition to PhoPQ, further layer(s) of regulation control *palA* expression.

We next aimed to more directly assess whether the *palA* expression pattern observed above is responsible for PalA’s signal-dependent role in PhoPQ-mediated regulation. To do this, we generated a low-copy expression vector that uses the highly tunable P_tet_ promoter to control *palA* expression to provide more physiologically relevant levels of *palA* expression that are independent of growth conditions. We then assessed PhoPQ activation in WT *pagC:lacZ* strains compared to Δ*palA* strains carrying this plasmid, using a range of inducer concentrations combined with either noninducing, CAMP, or low pH growth conditions. We found that, when expressed from the plasmid, *palA* impacted *pagC:lacZ* reporter levels in an almost identical manner for the CAMP and low pH conditions, and that there was no difference in the amount of *palA* induction needed to activate PhoPQ under these two signals (Fig. 3G). The observation that *palA* expression substantially increases PhoPQ activation under low pH conditions indicates that its limited effects under this condition in WT *S*. Typhi stems from its low expression levels, and supports the premise that it is the CAMP-dependent regulation of *palA* that underlies its enhanced role in PhoPQ regulation when this cue is present. Combining low levels of *palA* expression with either low pH or C18G triggered substantially higher *pagC:lacZ* activity than low pH alone, C18G alone, or saturating *palA* expression alone (Fig. 3G and H), suggesting that PalA works in concert with stimulatory growth conditions to further enhance PhoPQ activation. Our results also showed that higher aTc concentrations were needed for maximal PalA activity under noninducing conditions than under low pH or C18G conditions (Fig. 3G). This could be reflective of the reduced PhoP/PhoQ levels in *Salmonella* under noninducing conditions, or possibly that PalA more efficiently enhances PhoPQ activity when it is already in an activated state.

In summary, we find that *palA* expression is controlled by PhoPQ, as well as additional regulatory inputs that have not yet been identified. PalA is able to stimulate PhoPQ under various growth conditions, but since CAMPs stimulate its expression much more strongly than other PhoPQ-activating conditions, PalA’s effects are much more pronounced in this condition.

### PalA-mediated signaling is specific to the PhoPQ two-component system

**Fig 4:**
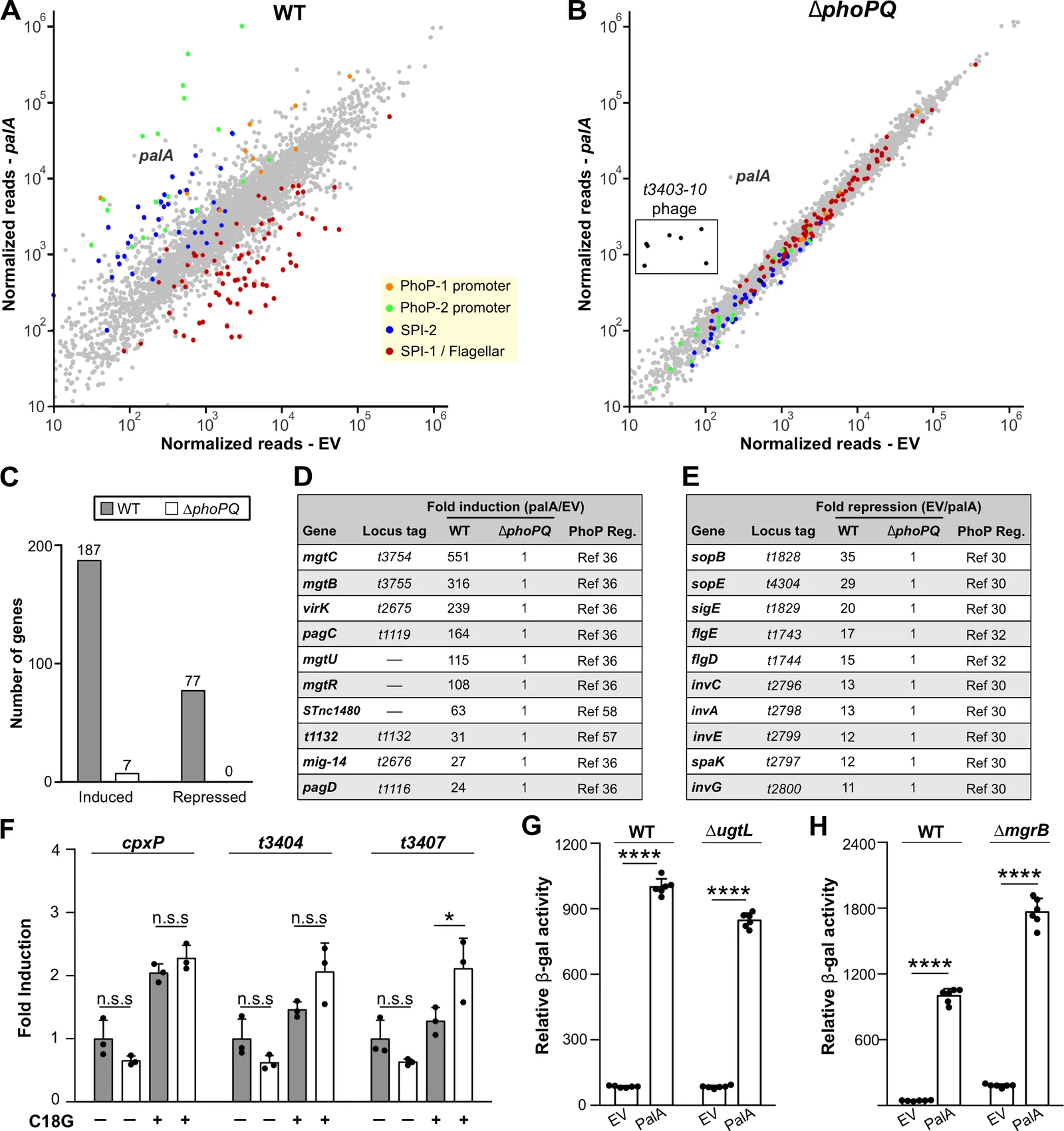
PalA expression has substantial and widespread effects on the expression of the PhoP regulon that are completely dependent upon PhoPQ. (**A and B**) RNA was isolated from WT or Δ*phoPQ S*. Typhi strains following 6 h of overexpression of *palA* or an empty vector (EV) control, and RNA-Seq was performed. Data points represent genes, and values represent the normalized average number of sequencing reads. Notable classes of genes are color-coded according to the legend; PhoP-1 and PhoP-2 distinctions are described in the main text. (**C**) The number of significant hits (>3-fold induction or repression by PalA overexpression compared to EV control across screen replicates) in the indicated strains. (**D and E**) The 10 most highly upregulated and downregulated genes by RNA-seq resulting from PalA overexpression. References provided under “PhoP Reg.” (30, 32, 36, 42, 43) represent one previous publication for each listed gene that provides evidence of PhoPQ regulation. (**F**) RT-qPCR was performed on WT (grey bars) or Δ*palA* (white bars) *S*. Typhi grown in TTIM with or without 5 µg/mL C18G for 2 h. Transcript levels of the indicated genes were normalized to *rpoB,* and fold induction compared to WT noninducing was calculated. (**G and H**) Relative β-galactosidase activity comparing WT *cdtB:lacZ* to the indicated deletion strains following 6 h of *palA* overexpression in LB + 0.2% arabinose to EV controls. For panels F–H, error bars represent standard deviation, and statistical significance between the indicated samples was calculated using two-tailed *t*-tests: n.s.s, not statistically significant; *P < 0.05, **P < 0.01, ***P < 0.001, ****P < 0.0001.

To examine the global impacts of PalA-mediated signaling, we grew *S*. Typhi in LB (noninducing) conditions and used RNA-seq to compare the transcriptome following PalA overexpression to empty vector controls in both a WT and a ∆*phoPQ* background (Fig. 4A and B; Table S1). Consistent with the large number of *Salmonella* genes directly or indirectly regulated by PhoPQ, we found that >250 genes were differentially expressed in the *palA* samples compared to the EV control in the WT strain (≥3-fold change in expression across replicates, *P* < 0.05; 187 genes upregulated by *palA* expression, 77 genes downregulated; see Fig. 4C, Tables S2 and S3). The list of differentially expressed genes is replete with known PhoP-regulated genes; for example, each of the 10 most highly induced and repressed genes by PalA has previously been shown to be regulated by PhoP (Fig. 4D and E). As expected, SPI-1 and motility genes were broadly repressed by PalA expression, whereas SPI-2 genes were broadly induced (Fig. 4A). To analyze PalA’s impact on the PhoP regulon, we leveraged previous PhoP promoter analyses to compile a set of characterized PhoP-regulated genes broadly divided into two categories: (i) where PhoP binds in predictable locations relative to sigma factor binding sites, and functions via a classic RNA polymerase recruitment mechanism (“PhoP-1,” orange in Fig. 4A), or (ii) those with variable numbers/locations/orientations of PhoP binding sites where PhoP functions—at least in part—by counteracting H–NS transcriptional silencing (“PhoP-2,” green in Fig. 4A) (36, 41). PhoP-1 promoters often regulate conserved (ancestral) members of the PhoP regulon, whereas PhoP-2 promoters predominantly regulate horizontally acquired genes that are often *Salmonella*-specific. We observed that, although both categories of promoters were generally induced by *palA* expression, genes under the control of PhoP-2 promoters were induced much more strongly in most cases (Fig. 4A). This is consistent with data above that showed that, in the presence of C18G, the Δ*palA* mutation impacted the expression of the horizontally acquired (PhoP-2) genes *cdtB* and *pagC* to a greater extent than the ancestral (PhoP-1) genes *phoP* and *rstA* (Fig. 2G). This is consistent with previous observations that PhoP-2 promoters are disproportionately impacted by the difference between modest and high levels of PhoPQ activity for reasons stemming from their generally lower affinity PhoP-binding sites and their more digital regulatory mechanisms (38). Not all known PhoP-regulated genes were impacted by PalA expression, which was expected since some such genes are subject to complex regulation. Regardless, these results demonstrate that *palA* overexpression broadly and robustly stimulates PhoP regulatory responses.

Importantly, while *palA* overexpression elicited widespread effects on transcription in the WT strain, it had almost no effect in the Δ*phoPQ* strain (Fig. 4B). In the Δ*phoPQ* strain, only seven genes were found to be differentially expressed in response to *palA* overexpression; *cpxP*, and several genes in an apparent operon (*t3403-t3410*) within a P2-family prophage (44). While it is not clear why the *t3403-t3410* phage genes are induced by PalA expression, *cpxP* is the most highly induced gene in the CpxRA regulon, which is stimulated by lipoprotein overexpression, suggesting that *cpxP* induction is likely an artifact of PalA overexpression (this experiment was performed prior to our development of the low-copy *palA* expression system) (45). To explore this issue further, we grew WT and Δ*palA* strains in TTIM+C18G and assessed the expression of *cpxP* and two representative genes from the *t3403-t3410* cluster using RT-qPCR (Fig. 4F). Consistent with the premise that the induction of these genes in our RNA-seq analysis was an artifact of overexpression, neither *cpxP* nor the *t3403*-t*3410* genes were induced by PalA under native conditions. In summary, PalA induces a strong and widespread transcriptional response on the PhoP regulon but has little-to-no effect on the transcriptome in the absence of PhoPQ. This precludes the possibility that PalA activates PhoPQ via pleiotropic effects on the cell or indirectly by acting on other global regulators that affect PhoPQ regulation. Instead, these data suggest that PalA’s activity is highly specific to stimulating the PhoPQ TCS.

Although our RNA-seq analysis indicated that PalA does not work indirectly through a global regulator, we reasoned that it could potentially activate PhoPQ indirectly through the known PhoPQ adaptor proteins MgrB or UgtL. To test this possibility, we overexpressed *palA* in WT, Δ*ugtL,* and Δ*mgrB cdtB:lacZ* strains and compared reporter activity to EV controls (Fig. 4G and H). As expected, the Δ*mgrB* strain exhibited higher background levels of *cdtB:lacZ* expression due to the absence of MgrB’s inhibitory effects on PhoPQ. However, PalA expression increased *cdtB:lacZ* levels substantially in both the Δ*mgrB* and Δ*ugtL* strains, indicating that *palA* does not require either of the known PhoPQ adaptor proteins for its activity.

### PalA localizes to the cytoplasmic membrane and interacts with PhoQ

**Fig 5:**
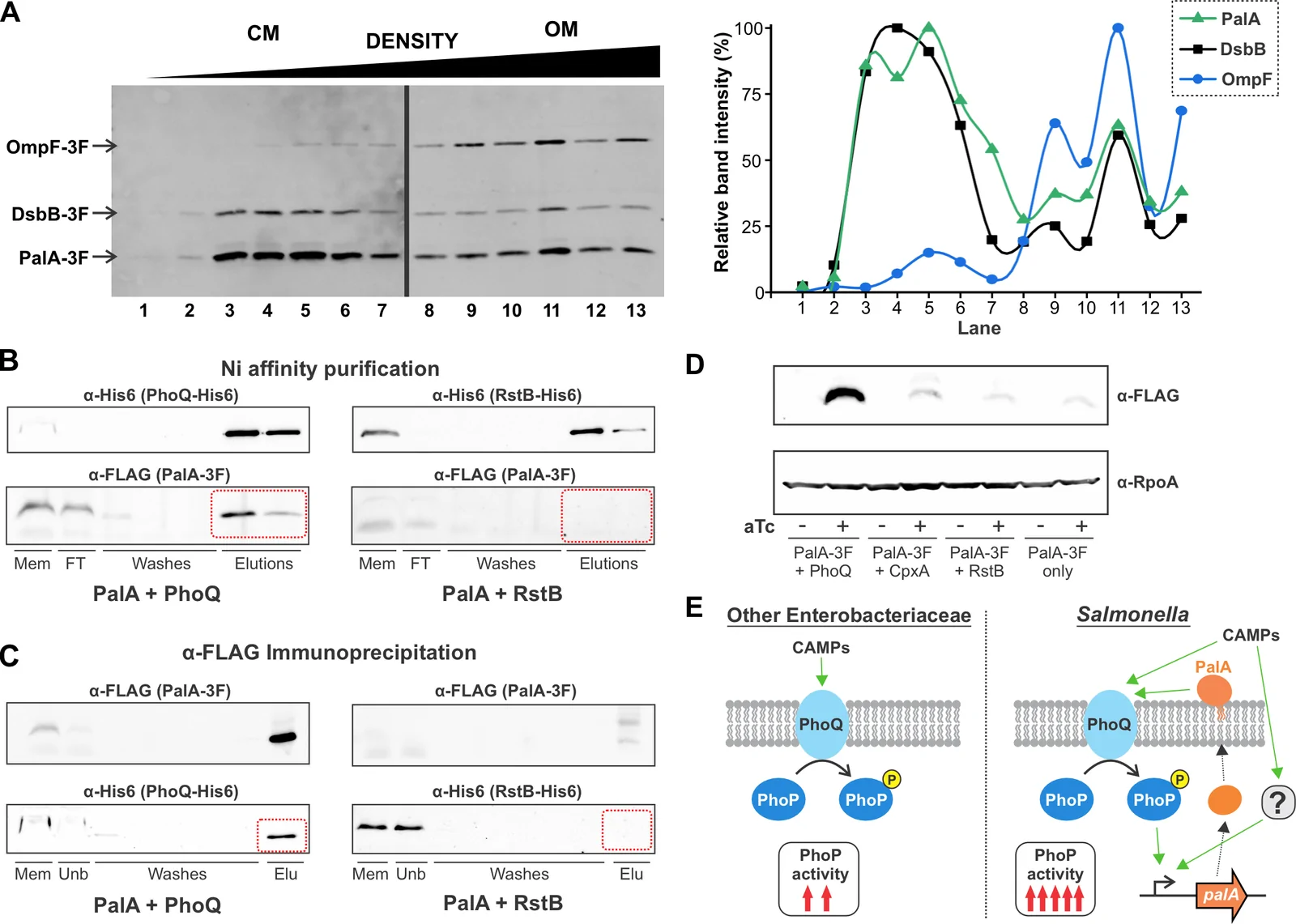
PalA localizes to the cytoplasmic membrane and interacts with PhoQ. (**A**) Membrane fractionation to track PalA localization. A *S*. Typhi *dsbB*-3F/*ompF*-3F strain carrying a low-copy plasmid expressing *palA*-3F was grown in LB + 200 ng/mL aTc for 6 h. Bacteria were then lysed, the membrane fraction isolated, and sucrose gradient fractionation was used to separate the CM and OM. Fractions were then analyzed by western blot using an α-FLAG antibody (left), and the band densities were calculated and analyzed (right). The black line between lanes 7/8 shows the dividing line between two different gels run in parallel. Results are representative of two independent experiments using this setup; similar results were observed with a higher copy PalA expression plasmid (Fig. S2). (**B**) Nickel column affinity purification of His6-tagged proteins, analyzing co-purification of PalA-3F with His6-tagged histidine kinases PhoQ (left) and RstB (right). *S*. Typhi Δ*palA*/*ΔphoPQ* strains transformed with a low-copy plasmid expressing PalA-3F and PhoQ-His6 or RstB-His6, as indicated, were grown overnight in LB containing 500 ng/mL aTc, at which point the cells were lysed, the membrane fraction isolated, and nickel affinity purification was performed. Membrane (mem), flow-through (FT), washes, and elutions were then analyzed by western blot using α-FLAG and α-His6 antibodies. (**C**) Immunoprecipitation (IP) of 3F-tagged PalA, analyzing co-purification of His6-tagged PhoQ (left) or RstB (right). Performed as described in panel B, except that α-FLAG IP was performed in place of nickel affinity purification. Unb, equivalent to FT, is the unbound material from the IP. Results for panels B and C are representative of two independent experiments with similar results; similar results were also observed using CpxA as the control histidine kinase (Fig. S4). Red boxes indicate the lanes assessing the co-purification of the target protein. (**D**) Western blot assessing PalA-3F levels in whole-cell lysates from Δ*palA*/Δ*phoPQ* strains carrying low-copy plasmids expressing the indicated proteins, grown as described in panel B. A parallel western blot using α-RpoA was used as a loading control. Results are representative of two independent experiments showing similar results. (**E**) Our model for enhanced PhoPQ activation by CAMPs in *Salmonella* due to PalA, compared to related organisms that do not encode PalA. The “?” represents unknown regulatory factor(s) that permits PhoPQ-mediated induction of *palA* expression in the presence of CAMPs, but not under low pH or low Mg^2+^ conditions.

Since the data above suggested that PalA is likely to be a direct activator of PhoPQ, we set out to determine whether PalA interacts with PhoPQ. In Gram-negative bacteria, some lipoproteins localize to the cytoplasmic membrane (CM), but many are translocated by the Lol system and localize to the outer membrane (OM) (46, 47). Given PalA’s small size, it would presumably need to localize to the CM in order to interact with PhoPQ, since it is too small to span the periplasmic space. To assess PalA localization, we used sucrose density gradient fractionation to separate the CM and OM and tracked PalA-3F localization relative to control proteins localized to the CM (DsbB) or OM (OmpF). Initial experiments using a higher copy overexpression system indicated that PalA localized predominantly or exclusively to the CM (Fig. S2); however, we reasoned that our overexpression system could conceivably overwhelm the Lol system, resulting in PalA mislocalization. Accordingly, we generated a new PalA-3F expression system using the low-copy aTc-inducible plasmid described above and repeated the fractionation experiment at significantly reduced PalA-3F expression levels that retained the ability to strongly activate PhoP (Fig. S3). We found that PalA’s fractionation pattern closely matched that of DsbB but was dissimilar from that of OmpF, indicating that PalA does indeed localize to the CM in *S*. Typhi (Fig. 5A).

Due to PalA’s CM localization and small size, we reasoned that it was more likely to interact with the CM-localized PhoQ than the cytoplasmic PhoP. To determine if PalA interacts with PhoQ, we used a co-immunoprecipitation/co-purification approach using C-terminal 3F-tagged PalA and 6xHis-tagged PhoQ co-expressed within *S*. Typhi. After isolating membrane fractions and performing nickel affinity column purification, we found that PalA-3F co-purified with PhoQ-His6, whereas PalA-3F did not co-purify with the histidine kinase RstB-His6 in analogous control experiments (Fig. 5B). Similarly, immunoprecipitation of PalA-3F using an α-FLAG antibody led to co-purification of PhoQ-His6, but not RstB-His6 (Fig. 5C). Similar experiments using PhoP in place of PhoQ, or using a second control histidine kinase, CpxA, also failed to detect an interaction with PalA (Fig. S4 and S5). Interestingly, we found that when PalA was co-expressed with PhoQ, it accumulated to higher levels than when expressed on its own or with the control histidine kinases CpxA and RstB (Fig. 5D), which suggests that interacting with PhoQ might stabilize PalA and reduce its turnover. To further investigate this, we analyzed PalA-3F levels expressed from the low-copy expression vector in a WT strain compared to a Δ*phoPQ* strain (Fig. S6). We observed a modest reduction in PalA-3F levels in the Δ*phoPQ* strain, suggesting that at native expression levels, PhoQ is able to enhance PalA levels in a manner that is independent of its role in regulating PalA transcription, consistent with a putative role in reducing PalA turnover. In summary, these experiments indicate that PalA localizes to the CM and interacts with PhoQ, suggesting that PalA directly interacts with PhoQ to stimulate PhoPQ activity. Our model for PalA’s role in *Salmonella* is presented in Fig. 5E.

### *Salmonella*’s PhoPQ system is evolutionarily adapted to promote its activation by PalA

**Fig 6:**
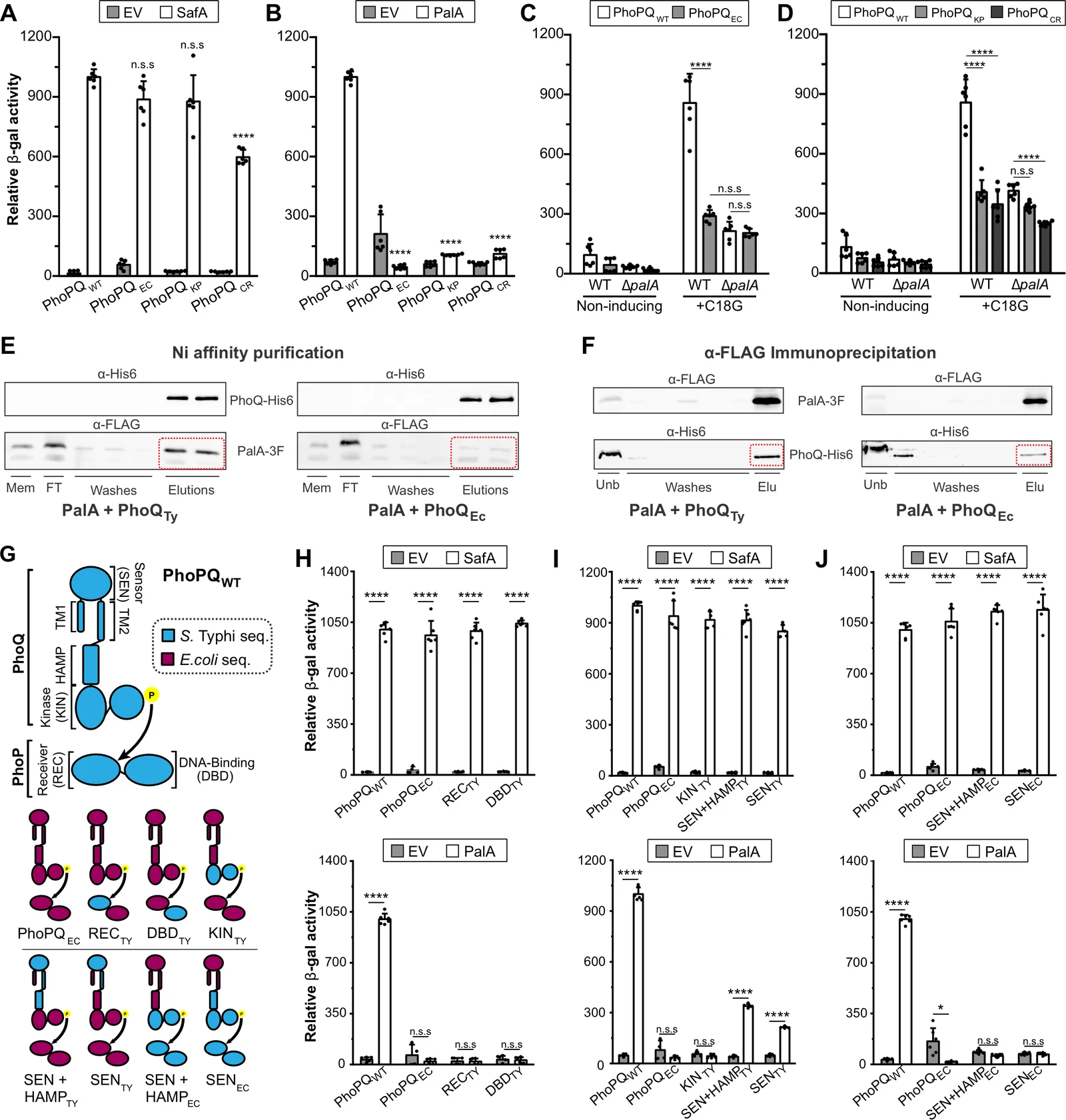
*Salmonella* PhoPQ has evolved to be activated by *palA*. (**A**) β-galactosidase activity following overexpression of *safA* compared to EV controls in *cdtB:lacZ* strains with either a WT *phoPQ* locus (*PhoPQ_WT_*) or the *PhoPQ_EC_*, *PhoPQ_KP_*, or *PhoPQ_CR_ phoPQ* locus, as indicated. Samples were grown for 6 h in LB + 0.2% arabinose prior to assay. (**B**) Similar to panel A, but *palA* overexpression. (**C and D**) Relative β-galactosidase activity in the indicated *cdtB:lacZ* strains grown in TTIM ± 5 µg/mL C18G for 6 h. (**E and F**) Co-purification/co-immunoprecipitation of PhoQ-His6 and PalA-3F comparing *S*. Typhi PhoQ (PhoQ_WT_) to *E. coli* PhoQ (PhoQ_EC_) using the methods described in the legend for Fig. 5. (**E**) Co-purification using nickel affinity purification of PhoQ-His6, (**F**) co-immunoprecipitation using an α-FLAG antibody to purify PalA-3F. Data are representative of at least two independent experiments with similar results. (**G**) Schematic representation of the chimeric *phoPQ* alleles assayed in panels H–J. TM, transmembrane segment, indicates the location of the CM, with the PhoQ sensor domain in the periplasm and the HAMP/kinase domains in the cytoplasm. (**H–J**) *safA* (top) or *palA* (bottom) was overexpressed compared to EV controls in the indicated *cdtB:lacZ* strains, shown schematically in panel G, and assayed for β-galactosidase activity after 6 h of growth in LB + 0.2% arabinose. For panels A–D and H–J, three biological replicates from two independent experiments (*n* = 6) were used; bars represent the average β-galactosidase activity, and error bars represent standard deviation. Statistical differences between indicated samples were calculated using one-way ANOVA with a Tukey’s post hoc test (A–D) or two-tailed *t*-tests (H–J). For panels A and B, statistical differences of samples compared to the PhoPQ_WT_ sample with SafA (A) or PalA (B) are shown. Statistical differences are represented as follows: n.s.s, not statistically significant; *P < 0.05, **P < 0.01, ***P < 0.001, ****P < 0.0001.

Since *palA* is unique to *Salmonella*, we reasoned that *Salmonella*’s PhoPQ system might have evolved to forge and optimize its functional relationship with PalA. To explore this possibility, we took advantage of a series of strains that we generated and tested in a previous study in which the *S*. Typhi *phoP* and *phoQ* genes were precisely replaced in the genome with the orthologous genes from the closely related species *E. coli* (PhoPQ_EC_), *Citrobacter rodentium* (PhoPQ_CR_), or *Klebsiella pneumoniae* (PhoPQ_KP_) (38). The PhoPQ systems in each of these strains were shown to be functional and to be activated by low Mg^2+^, low pH, C18G, and by infection of human epithelial cells (38). To determine whether these orthologous PhoPQ systems are also activated by PalA, we overexpressed *palA* in *cdtB:lacZ* reporter strains encoding the WT *S*. Typhi *phoPQ* locus (PhoPQ_WT_), as well as PhoPQ_EC_, PhoPQ_CR_, and PhoPQ_KP_. As a comparison, we similarly overexpressed the *E. coli*-specific PhoPQ adaptor protein SafA, which also activates PhoQ via direct interaction (27, 28). We found that SafA strongly activates the PhoPQ systems from all four species to a similar extent (Fig. 6A). In stark contrast, however, PalA overexpression strongly induced *cdtB:lacZ* expression in the strain encoding the *Salmonella* (PhoPQ_WT_) system (~14-fold), but not in the strains encoding orthologous PhoPQ systems (Fig. 6B; PhoPQ_CR_ and PhoPQ_KP_ were both induced <2-fold, PhoPQ_EC_ was repressed ~4-fold). Because orthologous PhoPQ systems are not effectively activated by PalA, we reasoned that the *S*. Typhi strains encoding them might exhibit reduced stimulation by C18G as a result. We thus compared *cdtB:lacZ* expression upon growth in TTIM+C18G for the strains carrying the various PhoPQ alleles, with and without a Δ*palA* mutation. We found that the PhoPQ_WT_ strain exhibited a markedly stronger response to C18G than strains encoding orthologous PhoPQs in the WT background, but that the C18G responses were similar across the different PhoPQ systems in the analogous Δ*palA* strains (Fig. 6C and D). All of the PhoPQ systems tested here, therefore, exhibit a similar intrinsic stimulation by C18G, but the strain encoding the *Salmonella* PhoPQ system is further activated by CAMPs due to its unique capacity to be further activated by PalA. This suggests that *Salmonella* PhoPQ has evolved to elicit an enhanced response to CAMPs compared to related species, not through its own intrinsic sensitivity to CAMPs, but rather through its interaction with PalA.

To further explore the specificity of PalA for *Salmonella*’s PhoPQ system, we used *E. coli* PhoPQ as a comparison. We first assessed the propensity of the two PhoPQ systems to interact with PalA by repeating our co-purification experiments, now comparing *Salmonella* and *E. coli* PhoQ (Fig. 6E and F). We found greater co-purification when using *Salmonella* PhoQ, suggesting that it interacts more strongly with PalA. However, *E. coli* PhoQ did co-purify with PalA, albeit to a lesser extent, suggesting a weak interaction. Consistent with this, similar amounts of PalA-3F were immunoprecipitated with the α-FLAG antibody when co-expressed with *Salmonella* and *E. coli* PhoQ (Fig. 6F, FLAG blots), which contrasts with what was observed with the control histidine kinase RstB (Fig. 5C, FLAG blots). This suggests that *E. coli* PhoQ might stabilize PalA to some degree, although this was not evident at native levels of PhoQ_EC_ expression (Fig. S6). Because *palA* overexpression was observed to significantly reduce *cdtB:lacZ* levels in the PhoPQ_EC_ strain (Fig. 6B), our data collectively suggest that *E. coli* PhoQ interacts weakly with PalA, and that this interaction may repress (rather than stimulate) its activity.

To identify which segment(s) of *Salmonella* PhoP and/or PhoQ confer its ability to be activated by PalA, we used a strategy wherein we began with the inactive PhoPQ_EC_ strain and restored the *S*. Typhi sequence to functional domains of PhoP or PhoQ (Fig. 6G) (38). Although *cdtB:lacZ* expression was similarly induced by *safA* expression in all of these strains, indicating their PhoPQ systems are functional, the only strains that were sensitive to *palA* expression were the one where PhoQ’s periplasmic sensor domain was restored to the *S*. Typhi sequence (Fig. 6H and I). It is noteworthy that the PalA response in these strains was not restored to WT levels, indicating that genetic differences beyond the sensor domain also contribute to PhoPQ’s stimulation by PalA. Taking the inverse approach, we found that introducing the *E. coli* PhoQ sensor domain sequence to an otherwise WT (*Salmonella*) PhoPQ was sufficient to completely abrogate PalA sensitivity, even though this strain was fully induced by SafA expression (Fig. 6J). Collectively, these results suggest that *Salmonella*’s PhoPQ system, most notably the PhoQ sensor domain, is evolutionarily adapted to promote its activation by PalA.

## DISCUSSION

*Salmonella*, which is among the most widespread and evolutionarily successful bacterial pathogens, has a unique virulence program driven by a large collection of virulence genes that are specific to this genus. Many such genes are regulated by the PhoPQ TCS. Although PhoPQ has a role in virulence gene expression for many pathogens, its role in *Salmonella* is fundamentally different, both in terms of the virulence program being regulated and the specific environmental niche(s) in which it is active. Here, we identify a *Salmonella*-specific evolutionary adaptation to the PhoPQ TCS, wherein the PalA adaptor protein enhances PhoPQ activation in a signal-specific manner. Under low Mg^2+^ conditions, a strong and evolutionarily conserved PhoPQ activating cue, PalA has little-to-no effect on PhoPQ activation. By contrast, in the presence of the CAMP C18G, PalA enhances PhoPQ activation, leading to markedly increased expression of PhoP-activated virulence genes. Elucidating the specific role of this regulatory node during infection will be a highly relevant avenue to address in future work. CAMPs are an important aspect of innate immunity, and PalA’s activity could potentially be important in various intracellular or extracellular host locations. PhoPQ is sensitive to numerous environmental inputs, and it is likely that multiple signals play a role in its activation *in vivo* and that the importance of different signals varies in different niches or at time points during infection. PalA activity does not require the presence of CAMPs, and we show that PalA is similarly able to enhance PhoPQ activation under low pH conditions when expressed at modest levels from a plasmid. PalA’s greater role in CAMP signaling therefore appears to be mostly or entirely due to the fact that its expression is higher under this condition than under low Mg^2+^ or low pH conditions. Deciphering the regulation of PalA could further clarify the spectrum of conditions that trigger its expression, which could shed light on the conditions/locations where it is utilized during infection.

A disproportionate number of the adaptor proteins that have been characterized to date relate to PhoPQ; it is unclear if this reflects the important and flexible role played by this TCS, or simply the fact that it is amongst the most highly studied TCS. Many adaptor proteins identified to date have limited phylogenetic distributions or have evolved to behave differently in different species. With regard to those related to PhoPQ biology, SafA is only found in *E. coli*, UgtL and PalA are only found in *Salmonella*, and the *E. coli* and *Salmonella* orthologs of MgrB exhibit different regulatory properties (26, 27, 38). Furthermore, PmrD, a PhoP-regulated adaptor protein that modulates the activity of the PmrAB TCS, has a limited phylogenetic distribution and exhibits different properties in different species (48, 49). Adaptor proteins therefore appear to be an important means by which the properties of a TCS can be modulated over evolution. In the present case, the evolutionary acquisition of PalA and its integration into *Salmonella*’s PhoPQ signaling network presumably reflect a need for greater PhoPQ activity under certain conditions encountered during infection. In this vein, it is noteworthy that the SPI-11 region of the genome where *palA* is found includes a large number of horizontally acquired PhoP-regulated genes (3, 50). Indeed, 13 out of 14 genes in a cluster that surrounds *palA* in the *S*. Typhi genome were identified by RNA-seq as being induced >3-fold by PalA overexpression (Fig. S7); this cluster includes some of the genes most highly induced by PalA in the genome (Fig. 4D). The genetic content of this region of the genome is variable in *Salmonella*. In some lineages—including both *S*. Typhi and the distantly related *S. bongori*—this gene cluster includes the typhoid toxin genes (51). In many lineages, including those important for human infection, such as *S*. Typhi and *S*. Typhimurium, this region encodes multiple proteins that are predicted to localize to the outer membrane, including PagC, which has been shown to be important for *Salmonella* survival in infection models (50, 52, 53). Given PalA’s role in further enhancing PhoPQ activity under inducing conditions, it is perhaps significant that many PhoP-induced genes are expressed at very high levels under infection conditions, which may be necessary for these virulence factors to function optimally. We propose that PalA forms a positive feedback loop with PhoPQ that is pronounced in the presence of CAMPs, which enhances the expression of important PhoP-activated genes involved in survival and pathogenesis, and that this has contributed to the expansion of PhoPQ’s function in *Salmonella*.

Because many adaptor proteins are very small and have a limited phylogenetic distribution, they can elude annotation and be missed in genetic screens. This study exemplifies this supposition; the *Salmonella* PhoPQ system had been the subject of intense study for decades prior to the present identification of PalA. Given the large number of TCS utilized by most bacteria, we hypothesize that, even in model organisms such as *S. enterica*, there might remain significant numbers of undiscovered adaptor proteins that modulate TCS activity in important ways.

## MATERIALS AND METHODS

### Plasmid and strain construction

Plasmids were generated using standard recombinant DNA techniques. Plasmids for the construction of mutant *S*. Typhi strains were based on the pSB890 plasmid as the vehicle for genetic transfer, as described previously (54, 55), wherein the mutation of interest was cloned between homology regions that flanked the mutation. Arabinose-inducible plasmids for overexpression of *palA* and *safA* were based on pSB3784, a tetracycline-resistant derivative of the pBAD30 plasmid (31), and were constructed by cloning an optimal ribosome binding site and the full coding sequence into this vector downstream of the arabinose-inducible promoter. The high-copy plasmid used for the initial localization experiments was also derived from pSB3784, where PalA had C-terminal 3×FLAG (DYKDHDGDYKDHDIDYKDDDDK) appended. The low-copy anhydrotetracycline-inducible plasmids used for the final localization experiment and the interaction (co-purification) experiments were derived from pWSK129, into which we cloned a fragment containing the TetR repressor and P_tet_ promoter from pFD152. PalA-3F, featuring an optimal ribosome binding site, was then cloned into this plasmid downstream of the P_tet_ promoter, and C-terminally His6-tagged histidine kinases (PhoQ, RstB, CpxA) were cloned downstream of PalA with their own optimal ribosome binding site. All plasmids were confirmed by sequencing and are shown in Table S4.

*S*. Typhi strains were derived from the wild-type strain ISP2825. Mutant strains were constructed using standard allelic exchange procedures with *E. coli* β-2163 Δ*nic35* as the conjugative donor strains (54, 55). For strains encoding *dsbB-3F* and *ompF-3F*, tags were introduced at the native genomic locus to the C-terminus of the indicated gene. For deletion mutations, the entire coding sequence of the indicated gene(s) was deleted from start codon to stop codon without introducing a scar. A complete list of strains used is found in Table S5.

### Growth conditions

Bacteria were cultured at 37°C while rotating or shaking in LB broth or in the indicated variations of TTIM—typhoid-toxin-inducing media. TTIM is a chemically defined medium based on N-minimal medium modified to optimally induce PhoPQ activity in *S*. Typhi (31). Noninducing TTIM contains 2 mM Mg^2+^, pH 7.2; low Mg^2+^ TTIM is altered to contain 10 µM Mg^2+^; low pH TTIM is altered to have a pH of 4.9; and TTIM+C18G contains 5 µg/mL of the CAMP C18G (Anaspec). To induce expression from plasmids, L-arabinose (BioShop Canada) or anhydrotetracycline (Thermo Scientific) was added as indicated.

### *In silico* analysis of *palA* sequences and distribution

To identify the distribution of *palA* coding sequences across and outside of *Salmonella* genomes, we used tBLASTn searches of the NCBI nr DNA sequence database using the *S*. Typhi PalA amino acid sequence as the query, cross-referencing this search with a similar search using the conserved RpoB protein as the query to infer PalA’s conservation. To identify PalA homologs outside of the *Salmonella* genus, we performed a similar search, filtering out *Salmonella* (taxid 590) results. The lipoprotein prediction programs SignalP 5.0 and DOLOP were used to infer that PalA is a putative lipoprotein and to predict its lipoprotein signal sequence (39, 56).

### β-galactosidase assays

For TTIM-based assays, overnight cultures in LB were pelleted and resuspended in equivalent volumes of TTIM, diluted 1/20 into fresh TTIM medium, and grown for 6 h at 37°C. For LB-based assays, overnight cultures were diluted 1/20 into LB + 0.2% arabinose or 1 mM IPTG, as indicated. To assay, 10 µL aliquots of culture were added to 90 µL of permeabilization buffer (100 mM Na_2_HPO_4_, 20 mM KCl, 2 mM MgSO_4_, 0.8 mg/mL hexadecyltrimethylammonium bromide, 0.4 mg/mL sodium deoxycholate, 5.4 µL/mL β-mercaptoethanol) and incubated for 20 min at room temperature. A volume of 600 µL of substrate buffer (60 mM Na_2_HPO_4_, 40 mM NaH_2_PO_4_, 2.7 µL/mL β-mercaptoethanol, 1 mg/mL ONPG) was added to start the reactions, and once reactions developed a yellow color, 700 µL of quench buffer (1 M Na_2_CO_3_) was added to stop the reaction. Samples were then centrifuged for 5 min at 20,000 × *g* to remove cell debris before recording OD_420_ measurements. β-galactosidase activity (in Miller units) was calculated as follows: (1,000 × OD_420_)/(reaction time [min] × culture vol assayed [mL] × OD_600_ [culture]). Where indicated, for overexpression assays, the average value of the wild-type PalA or SafA overexpression samples in the WT strain was scaled to a value of 1,000, and all data points were scaled by the same factor. In TTIM-based assays, where indicated, the wild-type samples in the inducing condition were used as the reference point and were similarly normalized to 1,000.

### Western blot analyses

For western blot analysis of protein levels in whole-cell lysates, strains were grown as indicated, after which the OD_600_ was measured to equalize gel loading, and samples were pelleted and resuspended in 2× SDS-buffer. SDS-PAGE and western blots were run according to standard protocols. Nitrocellulose membranes (Bio-Rad) were blocked using 5% non-fat milk in tris-buffered saline (TBS) for at least 45 min at room temperature and then incubated overnight at 4°C with the indicated primary antibody. The primary antibodies used here were monoclonal α-FLAG M2 (mouse, 1:5,000, Sigma-Aldrich, F3165), monoclonal α-RpoA (mouse, 1:5,000, BioLegend, 663104), and polyclonal α-His6 (rabbit, 1:20,000, Invitrogen, PIPA1983B). After repeated washes in TBS-1% Tween-20 (TBST), membranes were incubated with secondary antibody for 2 h at room temperature covered from light using 1:15,000 IRDye 680RD Goat anti-Mouse IgG (Li-Cor, 926-68070) or IRDye 800CW Goat anti-rabbit IgG (Li-Cor, 926-32211). After repeated washes in TBST and TBS, membranes were visualized with a Bio-Rad ChemiDoc imaging system. The specificity of the 3×FLAG and His6 signals detected was confirmed using western blots with lysates from otherwise identical strains lacking the 3×FLAG/His6-tagged protein in question.

Band quantification was performed using the ImageJ image processing and analysis software (https://imagej.net/ij/). For experiments quantifying protein levels under different conditions/strains, the band intensities of each lane for each band from at least three independent experiments were determined, removing background intensity. The highest intensity band in each experiment for each protein was used to normalize the relative abundance of other bands. The relative abundance across at least three experiments was quantified and averaged.

### RNA isolation and quantitative RT-qPCR

Overnight cultures were resuspended and grown for 6 h in TTIM or LB + 0.2% arabinose as appropriate. Bacterial pellets were collected and stored at −80°C. Total RNA was collected from frozen bacterial pellets using hot acid phenol:chloroform extractions. Pellets were resuspended in TES (10 mM Tris HCl pH 7 to pH 8, 10 mM EDTA, 0.5% SDS), and two to four sequential extractions were performed using acid phenol:chloroform (Ambion), followed by one chloroform extraction. RNA was precipitated using isopropanol and glycogen (Invitrogen), and pellets were washed twice with 70% ice-cold ethanol before the air-dried RNA pellets were resuspended in RNase-free H_2_O. Purified RNA samples were treated with DNase I (Invitrogen) and precipitated using ethanol and glycogen. One microgram of RNA was used as a template for cDNA synthesis using Superscript II (Thermo Fisher) according to the manufacturer’s protocol for random hexamer priming. qPCR was performed using SYBR-green supermix and the 7500 Fast Real Time PCR Machine (Applied Biosystems) using a 40-cycle, two-step PCR program, followed by a melting curve, which was used to assess for uniform PCR products. Transcript levels were determined using primers specific for the indicated genes and serially diluted *S*. Typhi genomic DNA was used to generate standard curves for each primer set to facilitate the standardization of the data. The transcript levels for test genes were normalized to the constitutively expressed housekeeping gene *rpoB*. Fold induction levels were determined by dividing the transcript levels of each sample by the transcript levels of the average of the noninduced wild-type sample.

### RNA-seq

Overnight cultures carrying the *palA* overexpression plasmid or an empty vector control were grown for 6 h in LB + 0.02% arabinose. Total RNA was collected as described above, and RNA integrity was determined using the RNA 6000 Nano assay with the Agilent 2100 Bioanalyzer system. Sequencing was performed using the SeqCenter (Pittsburgh, PA, USA) with rRNA depletion service. The Illumina Stranded Total RNA Prep with Ribo-Zero Plus and 10 bp unique dual indices was used to generate libraries. Illumina NovaSeq X Plus was used to sequence 150 bp paired-end reads. Demultiplexing, quality control, and adapter trimming were performed with bcl-convert (v4.1.5) (Illumina). Sequencing reads were mapped to the *S*. Typhi Ty2 strain using the Bowtie2 and featureCounts programs on Galaxy. rRNAs and tRNAs were removed. To normalize read counts between samples, for each individual sample, the number of sequencing reads was scaled for each gene to produce an average of 10,000 reads/gene. The complete experiment, from growth and RNA isolation through analysis, was completed twice independently, with each sample in singlicate in each experiment. The ratio of the read numbers in the PalA overexpression sample to the empty vector sample was calculated, and genes with fold induction or repression values equal to or greater than 3 in both experimental replicates were considered to be significant hits. A value of 200 (2% of the average read value) was added to the normalized read numbers when calculating fold-induction and fold-repression values to reduce spurious results for low-expression genes stemming from transcriptional noise and sampling error.

### Analysis of protein localization

Membrane fractionation by sucrose density ultracentrifugation was performed as described previously (57). A *dsbB-3F + ompF-3F S*. Typhi strain carrying a plasmid that expresses *palA-3F* was grown in LB + 200 ng/mL aTc (for the low-copy plasmid) or 0.02% arabinose (for the high-copy plasmid) for 6 h in 250 mL LB. The cells were cooled and pelleted at 4,000 × *g* 4°C for 10 min, then stored at −80°C. Cells were resuspended in 20 mL of 10 mM Tris-HCl, pH 7.5. Cells were pelleted and resuspended in 5 mL Tris-sucrose buffer (10 mM Tris-HCl, pH 7.5, 0.75 M sucrose, 100 µg/mL gentamicin). Lysozyme (50 µg/mL), PMSF (2 mM), and 10 mL of 1.65 mM EDTA pH 7.5 were added to destabilize the outer membrane. Following incubation on ice for 10–15 min, cells were lysed using up to four passes through an Emulsiflex B15 High Pressure Homogenizer (Avestin) and centrifuged at 15,000 × *g* for 20 min to remove the cell debris. The supernatant was pelleted by ultracentrifugation at 38,000 rpm for 45 min at 4°C in a 70.1 Ti fixed-angle rotor (Beckman-Coulter). Total membrane pellets were resuspended in TES buffer (3.3 mM Tris-HCl, pH 7.5, 1.1 mM ETA, 0.25 M sucrose), the ultracentrifugation was repeated, and the membrane pellet was broken up using a sterile pipette tip and resuspended in 500 µL of 30% sucrose in 5 mM EDTA, pH 7.5. The membranes were applied to a six-step continuous sucrose gradient consisting of 4 mL of 63%, 55%, 50%, 45%, 40%, and 35% sucrose in 5 mM EDTA, pH 7.5. The membranes were separated across the gradient by ultracentrifugation at 40,000 rpm for 17 h at 4°C. A volume of 2 mL fractions was collected from the top of the gradient, and a portion of each fraction was combined with equivalent volumes of 2× SDS-buffer and used for western blot analysis.

### Interaction experiments

The methods for isolating membrane proteins were adapted from reference 58. Strains were grown overnight, subcultured 1/20 into 100 mL LB + 500 ng/mL aTc, and incubated at 37°C for 24 h while shaking at 220 RPM. Bacteria were collected by centrifugation at 4°C, 5,000 × *g* for 6 min, and resuspended in 10 mL of Tris/NaCl buffer (50 mM Tris, pH 7.5, 150 mM NaCl) supplemented with lysozyme (20 mg/mL) and cOmplete protease inhibitor cocktail. Cells were incubated at 4°C for 30 min prior to lysing using an EmulsiFlex-B15 High Pressure Homogenizer (Avestin). Lysates were clarified by centrifugation at 4°C, 7,000 × *g* for 30 min, discarding the pellet. To generate membrane fractions, clarified lysates were further ultracentrifuged at 4°C, 38,000 RPM, for 1 h in a Beckman Type 70Ti rotor. The pellets were resuspended in 650 μL of membrane solubilization buffer (50 mM Tris, pH 7.5, 150 mM NaCl, 1% n-dodecyl-β-D-maltopyranoside) and solubilized at 4°C for 30 min while rotating end-over-end. After solubilizing, aliquots were collected as the unpurified membrane samples, and the remaining sample was purified using Ni-NTA spin columns (NEB, S1427L) or immunoprecipitated with anti-FLAG M2 Affinity Gel (Sigma-Aldrich, A2220) according to the manufacturer’s instructions. Briefly, Ni Spin columns were equilibrated with Tris/NaCl buffer, loaded with 300 μL of solubilized membrane, and incubated at 4°C for 2 h while rotating. Columns were washed three times with Tris/NaCl buffer containing 5 mM imidazole and eluted twice with Tris/NaCl buffer containing 500 mM imidazole. Aliquots were collected after each spin and analyzed by western blot. For M2-FLAG IPs, solubilized membranes were incubated with agarose beads in a similar manner to the Ni columns. Beads were washed five times with Tris/NaCl buffer and eluted by incubating with SDS-PAGE loading buffer lacking β-mercaptoethanol and bromothymol blue at 98°C and 600 RPM in an orbital shaking heat block for 3 min. Eluates (supernatants) were separated from the beads and mixed with β-mercaptoethanol and bromothymol blue to generate the elution fractions.

## SUPPLEMENTAL MATERIAL

Supplemental material mbio.01070-26-s0001.pdf Supplemental tables and figures.

Supplemental tables mbio.01070-26-s0002.xlsx Tables S1–S3.

ASM does not own the copyrights to Supplemental Material that may be linked to, or accessed through, an article. The authors have granted ASM a non-exclusive, world-wide license to publish the Supplemental Material files. Please contact the corresponding author directly for reuse.
