# Supplementary material for: A Salmonella-specific protein enhances the antimicrobial peptide response of the PhoPQ two-component system
Source: mBio. 2026 Oct 9;17(11):e01070-26. doi: 10.1128/mbio.01070-26 (PMC13753844; doi:10.1128/mbio.01070-26)
Supplement: Supplementary file 1 [file mbio.01070-26-s0001.pdf]

Tables S1-S3 are presented in a separate .xlsx file.

Table S4: List of plasmids used in this study:

**Plasmid**	**Relevant features**	**Reference**
pSB3784	pBAD-based over-expression plasmid (empty vector)	PMID: 29324231
pCCF0091	pSB3784 + *sty1874-sty1869*	This study
pCCF0092	pSB3784 + *sty1873-sty1869*	This study
pSB4701	pSB3784 + *palA*	This study
pCCF0134	pSB3784 + *safA*	PMID: 40127991
pSB890	Mutagenesis vector	PMID: 7997169
pSB4799	pSB890-Δ*palA*. Used to delete *palA*	This study
pCCF0220	pSB890-*palA*. Used to restore *palA* in genome of Δ*palA* strains	This study
pCCF0078	pSB890-*ompF*-3F. Used to add 3F tag to OmpF in genome	This study
pCCF0216	pSB890-*dsbB*-3F. Used to add 3F tag to DsbB in genome	This study
pCCF0224	pSB890-*phoQ* SEN+TM_{EC}. Used to make *phoQ* SEN+TM_{EC} strain	This study
pCCF0306	pSB890-*phoQ* SEN_{EC}. Used to make *phoQ* SEN_{EC} strain	This study
pCCF0356	pWSK129 + P_{tet}. Low copy plasmid with P_{tet} promoter (empty vector) for tunable expression	This study
pCCF0252	pCCF0356 + *palA*	This study
pCCF0366	pCCF0356 + *palA*-3F	This study
pCCF0233	pCCF0356 + *palA*-3F + *phoQ*-His6	This study
pCCF0249	pCCF0356 + *palA*-3F + *cpxA*-His6	This study
pCCF0248	pCCF0356 + *palA*-3F + *rstB*-His6	This study
pCCF0253	pCCF0356 + *palA*-3F + *phoP*-His6	This study
pCCF0247	pCCF0356 + *palA*-3F + $phoQ_{EC}$-His6	This study

Table S5: List of strains used in this study:

**Strain**	**Genotype (relative to ISP2825)**	**Reference**
ISP2825	Wild-type *Salmonella enterica* serovar Typhi strain	PMID: 1879916
SB2913	Δ*phoPQ*	PMID: 29324231
SB2619	Δ*palA*	This study
SB2613	*cdtB:lacZ*	PMID: 29324231
SB2626	*cdtB:lacZ,* Δ*phoPQ*	PMID: 29324231
CCF0070	*cdtB:lacZ,* Δ*palA*	This study
CCF0158	*cdtB:lacZ,* Δ*palA + palA* complement	This study
CCF0087	*cdtB:lacZ,* Δ*mgrB*	PMID: 40127991
SB2624	*cdtB:lacZ,* Δ*ugtL*	PMID: 40127991
CCF0021	*cdtB:lacZ,* $phoPQ_{EC}$	PMID: 40127991
CCF0085	*cdtB:lacZ,* $phoPQ_{CR}$	PMID: 40127991
CCF0092	*cdtB:lacZ,* $phoPQ_{KP}$	PMID: 40127991
CCF0024	*cdtB:lacZ,* $phoPQ_{EC}$ SEN_{TY}	PMID: 40127991

CCF0026	*cdtB:lacZ, phoPQ$_{EC}$* DBD$_{TY}$	PMID: 40127991
CCF0027	*cdtB:lacZ, phoPQ$_{EC}$* REC$_{TY}$	PMID: 40127991
CCF0029	*cdtB:lacZ, phoPQ$_{EC}$* SEN+HAMP$_{TY}$	PMID: 40127991
CCF0030	*cdtB:lacZ, phoPQ$_{EC}$* KIN$_{TY}$	PMID: 40127991
CCF0201	*cdtB:lacZ, phoQ* SEN+TM$_{EC}$	This study
CCF0202	*cdtB:lacZ, phoQ* SEN$_{EC}$	This study
CCF0200	*cdtB:lacZ, phoPQ$_{EC}$*, Δ*palA*	This study
CCF0206	*cdtB:lacZ, phoPQ$_{CR}$*, Δ*palA*	This study
CCF0207	*cdtB:lacZ, phoPQ$_{KP}$*, Δ*palA*	This study
SB2799	*pagC:lacZ*	PMID: 40127991
CCF0074	*pagC:lacZ,* Δ*phoPQ*	PMID: 40127991
CCF0071	*pagC:lacZ,* Δ*palA*	This study
CCF0159	*pagC:lacZ,* Δ*palA + palA* complement	This study
SB3219	*cdtB-3F, pltC-3F*	PMID: 31417089
SB3229	*cdtB-3F, pltC-3F,* Δ*phoPQ*	PMID: 31417089
CCF0157	*cdtB-3F, pltC-3F,* Δ*palA*	This study
CCF0154	*ompF-3F, dsbB-3F*	This study
CCF0342	Δ*phoPQ*, Δ*palA*	This study

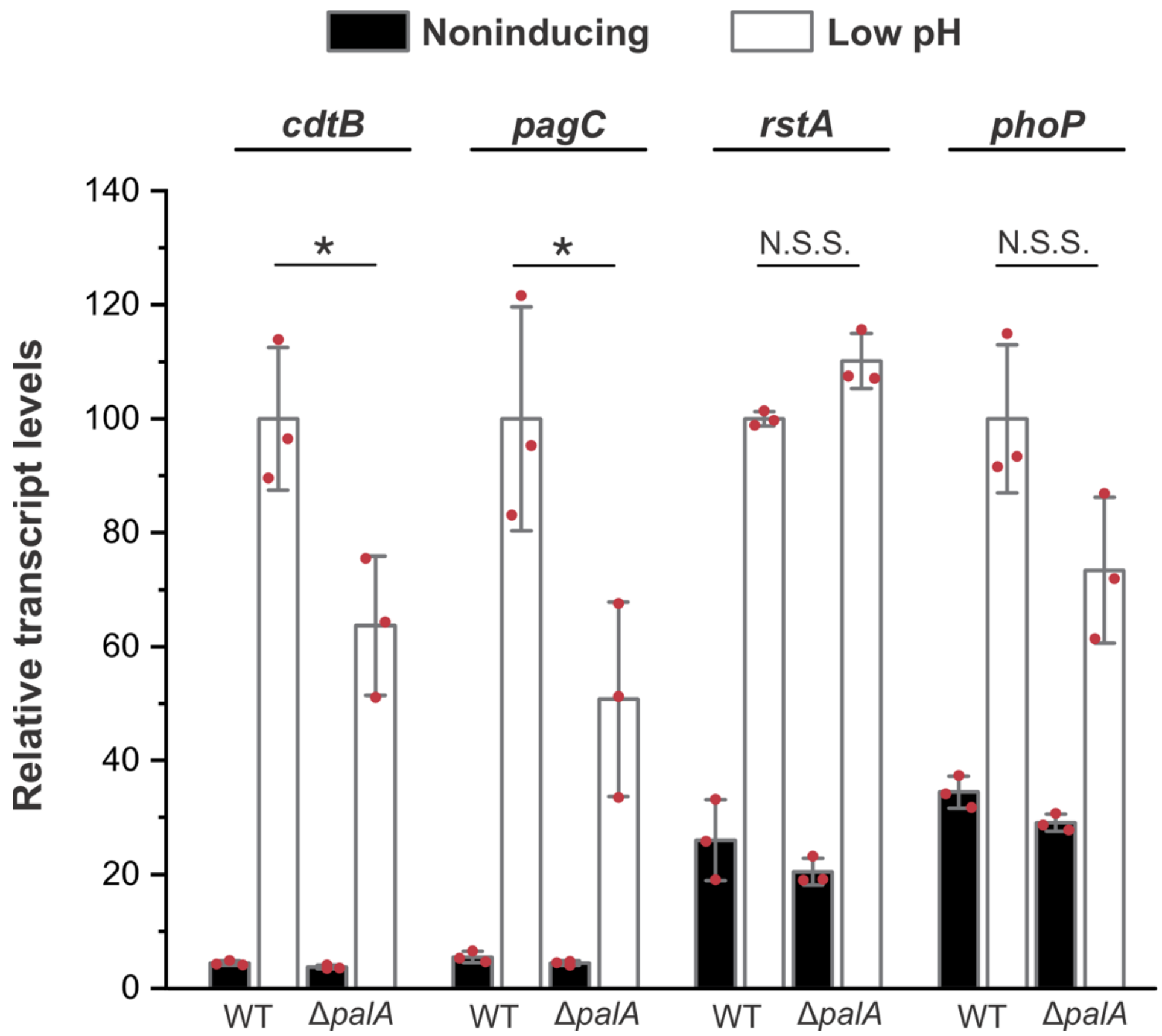

Fig. S1: PalA has a minor impact on the transcript levels of PhoP-activated genes upon growth in low pH conditions. WT or Δ*palA S*. Typhi were grown in TTIM 2 mM Mg^{2+} at a pH of either 7.2 (noninducing) or 4.9 (inducing) for 2 hr. RNA was extracted and RT-qPCR was used to quantify transcript levels of the indicated genes, normalized to the control gene *rpoB*. Relative transcript levels were then derived by scaling the average value for each gene in the WT low pH samples to be 100, and then scaling all other values for that gene proportionally. Data points represent three biological replicates for each sample and bars represent the average value. Error bars represent standard deviation and statistical differences between the indicated samples were calculated using two-tailed t-tests: N.S.S., not statistically significant, $^*p<0.05$.

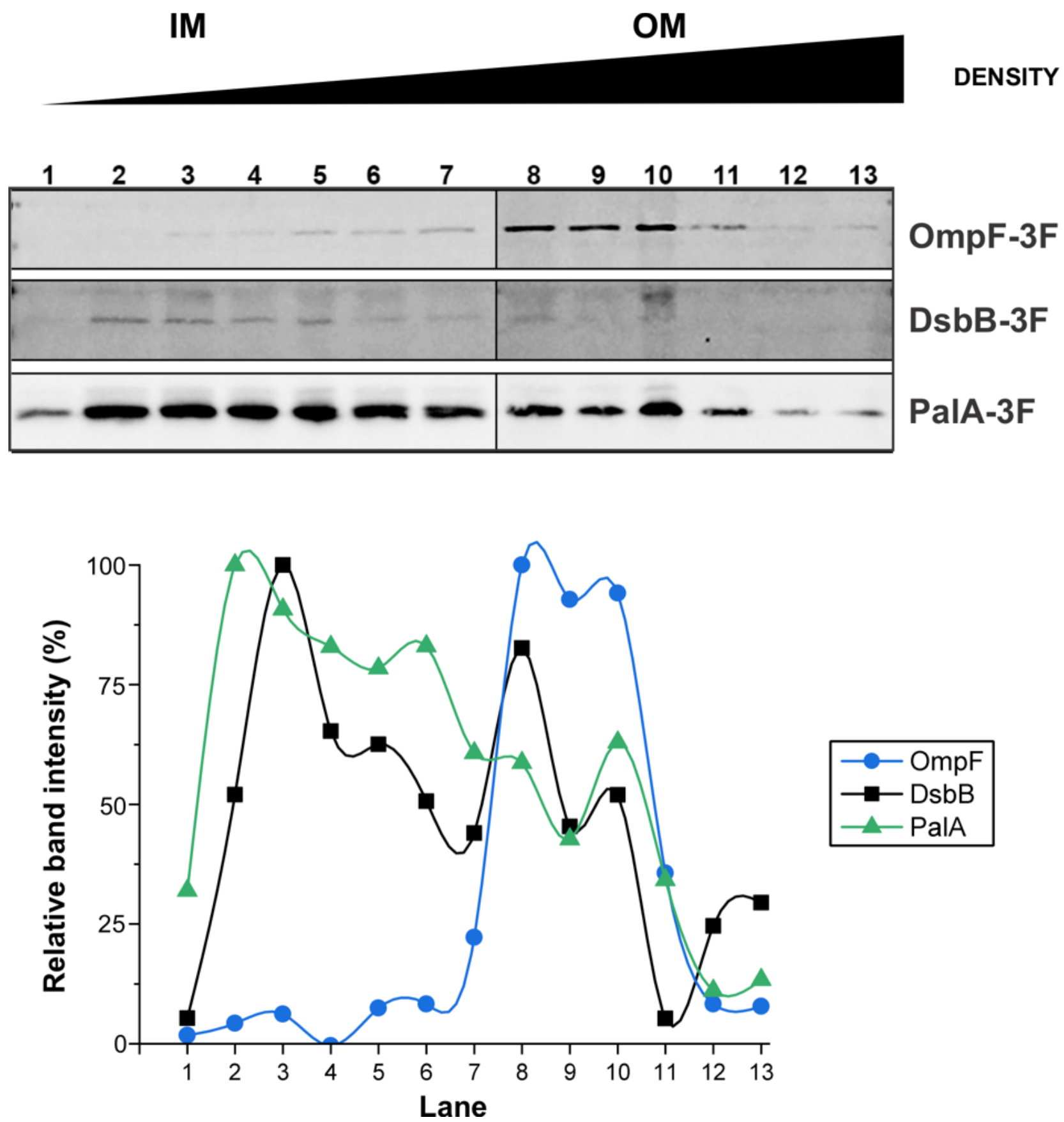

Fig. S2: *palA* over-expressed from the pBAD-based plasmid localizes to the outer membrane. Membrane fractionation to track PalA localization. A S. Typhi dsbB-3F/ompF-3F strain carrying medium copy pBAD-based plasmid expressing palA-3F was grown in LB + 200 0.02% arabinose for 6 hours. Bacteria were then lysed, the membrane fraction isolated and sucrose gradient fractionation was used to separate the CM and OM. Fractions were then analyzed by western blot using an α-FLAG antibody (top) and the band densities were calculated and analyzed (bottom). The black line between lanes 7/8 shows the dividing line between two different gels run in parallel the accommodate the large number of samples. Blots for the different proteins are presented in separate panels to optimize signal intensity for each protein individually. Results representative of three independent experiments that showed similar results.

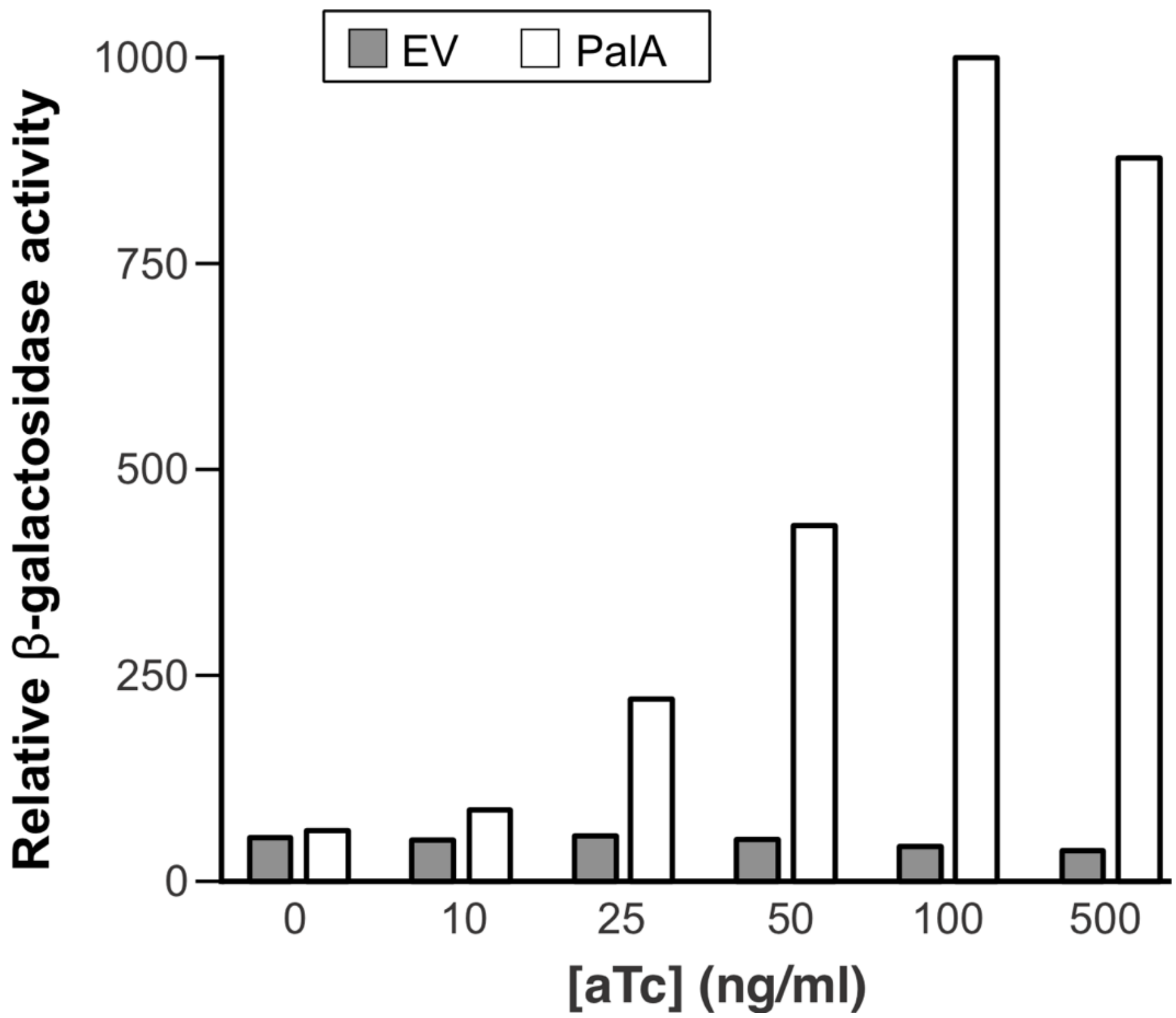

Fig. S3: Activity of *palA-3F* across inducer concentrations when expressed from our low copy, aTc-inducible expression plasmid. The pagC:lacZ strain carrying either the low-copy aTc inducible plasmid expressing palA-3F or an empty vector (EV) control was growth for 6 hr in LB contraining the indicated concentration of aTc, and then assayed for β-galactosidase activity. Bars represent the normalized activity levels compared to the concentration showing the highest activity.

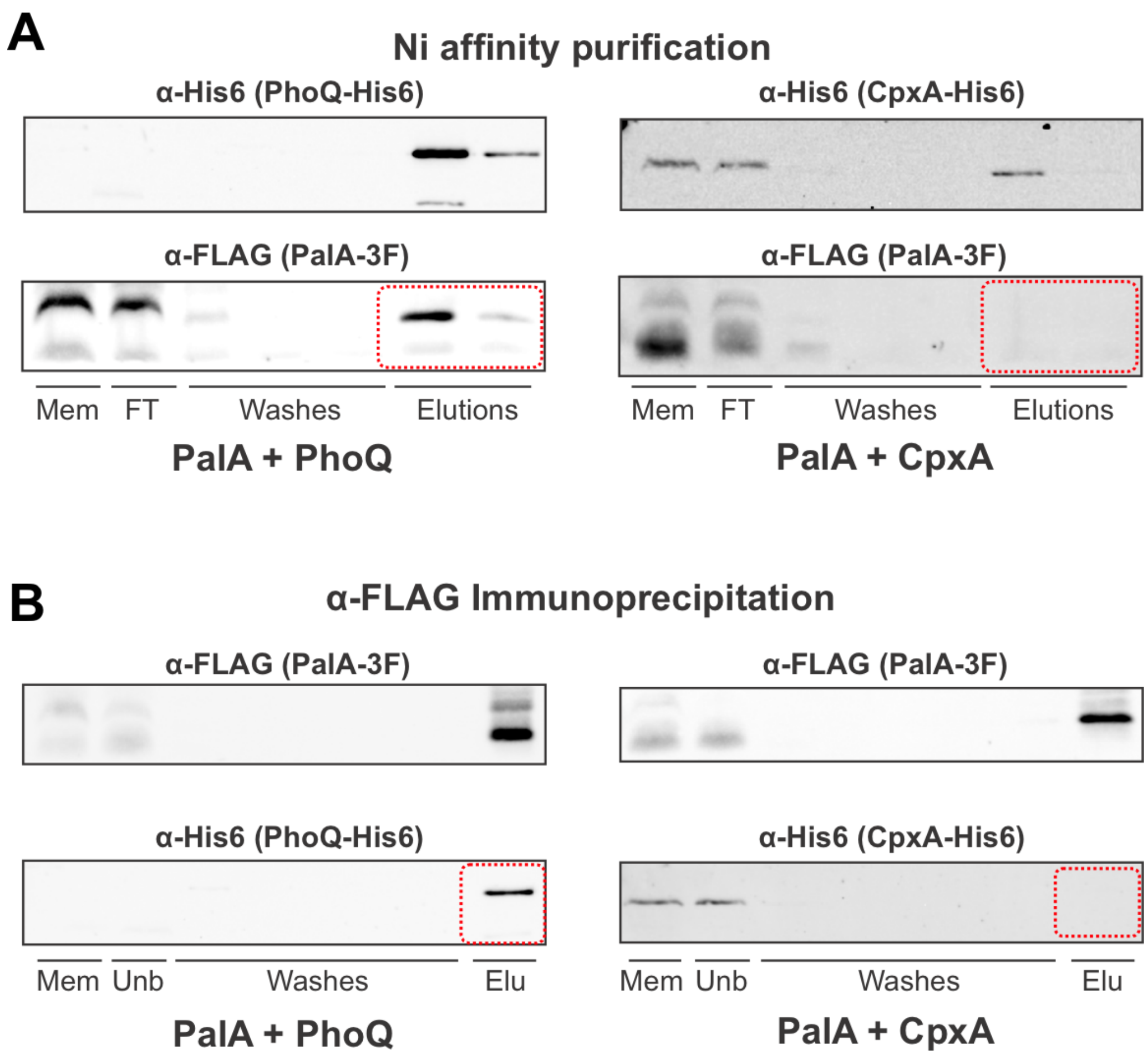

Fig S4: PalA does not interact with the control histidine kinase CpxA. A) Nickel column affinity purification of His6-tagged proteins, analyzing co-purification of PalA-3F with His6-tagged histidine kinases PhoQ (left) and CpxA (right). A *S*. Typhi Δ*palA* / Δ*phoPQ* strains was transformed with a low copy plasmid expressing PalA-3F and PhoQ-His6 or RstB-His6 as indicated, and was grown overnight in LB containing 500 ng/m of aTc, at which point the cells were lysed, the membrane fraction isolated, and nickel affinity purification was performed. Membrane (mem), flow through (FT), washes and elutions were then analyzed by western blot using α-FLAG and α-His6 antibodies. **B)** Immunoprecipitation (IP) of 3F-tagged PalA, analyzing co-purification of His6-tagged PhoQ (left) or CpxA (right). Performed as described in (A), except that α-FLAG IP was performed in place of nickel affinity purification. Unb, equivalent to FT, is the unbound material from the IP. Red boxes indicate the lanes assessing the co-purification of the target protein.

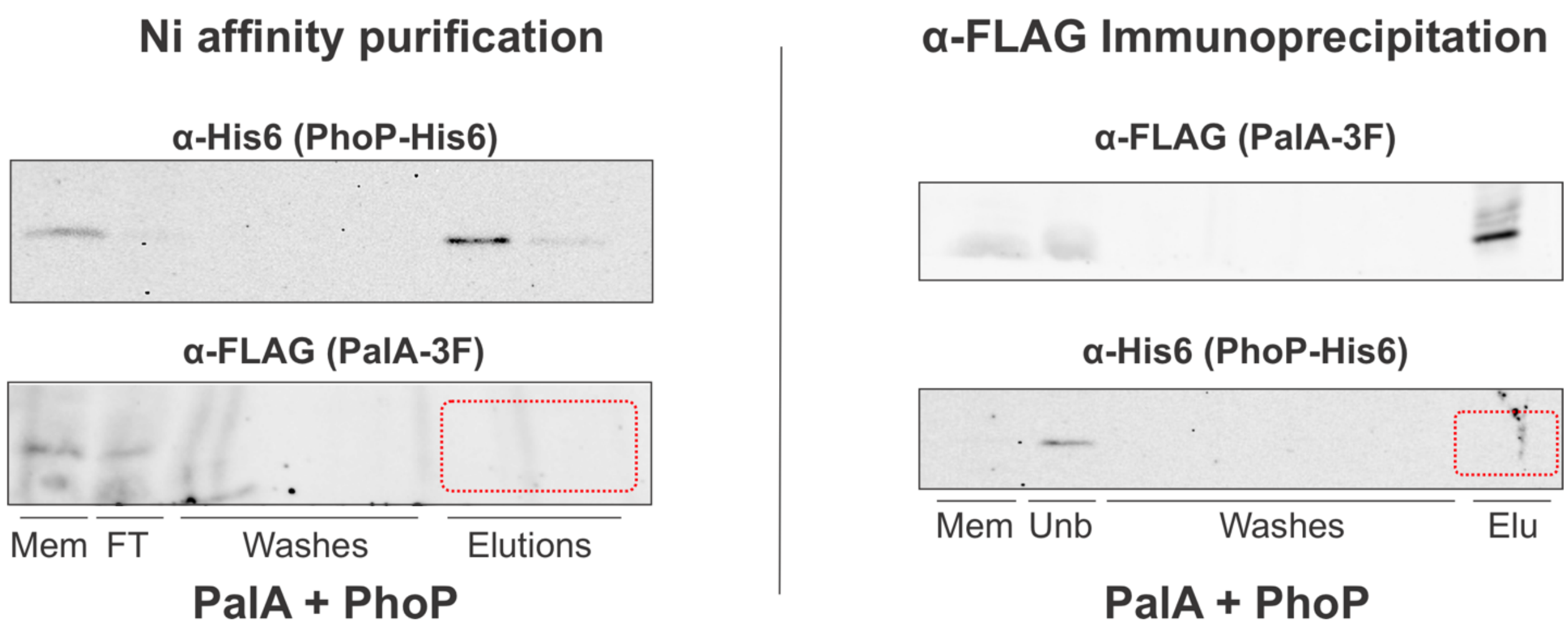

Fig S5: PalA does not interact with PhoP. Left, Nickel column affinity purification of His6-tagged PhoP, analyzing possible co-purification of PalA-3F. A *S*. Typhi Δ*palA* / Δ*phoPQ* strain was transformed with a low copy plasmid expressing PalA-3F and PhoP-His6 and grown overnight in LB containing 500 ng/m of aTc, at which point the cells were lysed, the membrane fraction isolated, and nickel affinity purification was performed. Membrane (mem), flow through (FT), washes and elutions were then analyzed by western blot using α-FLAG and α-His6 antibodies. Right, immunoprecipitation (IP) of 3F-tagged PalA, analyzing possible co-purification of His6-tagged PhoP. Performed as described in (A), except that α-FLAG IP was performed in place of nickel affinity purification. Unb, equivalent to FT, is the unbound material from the IP. Red boxes indicate the lanes assessing the co-purification of the target protein.

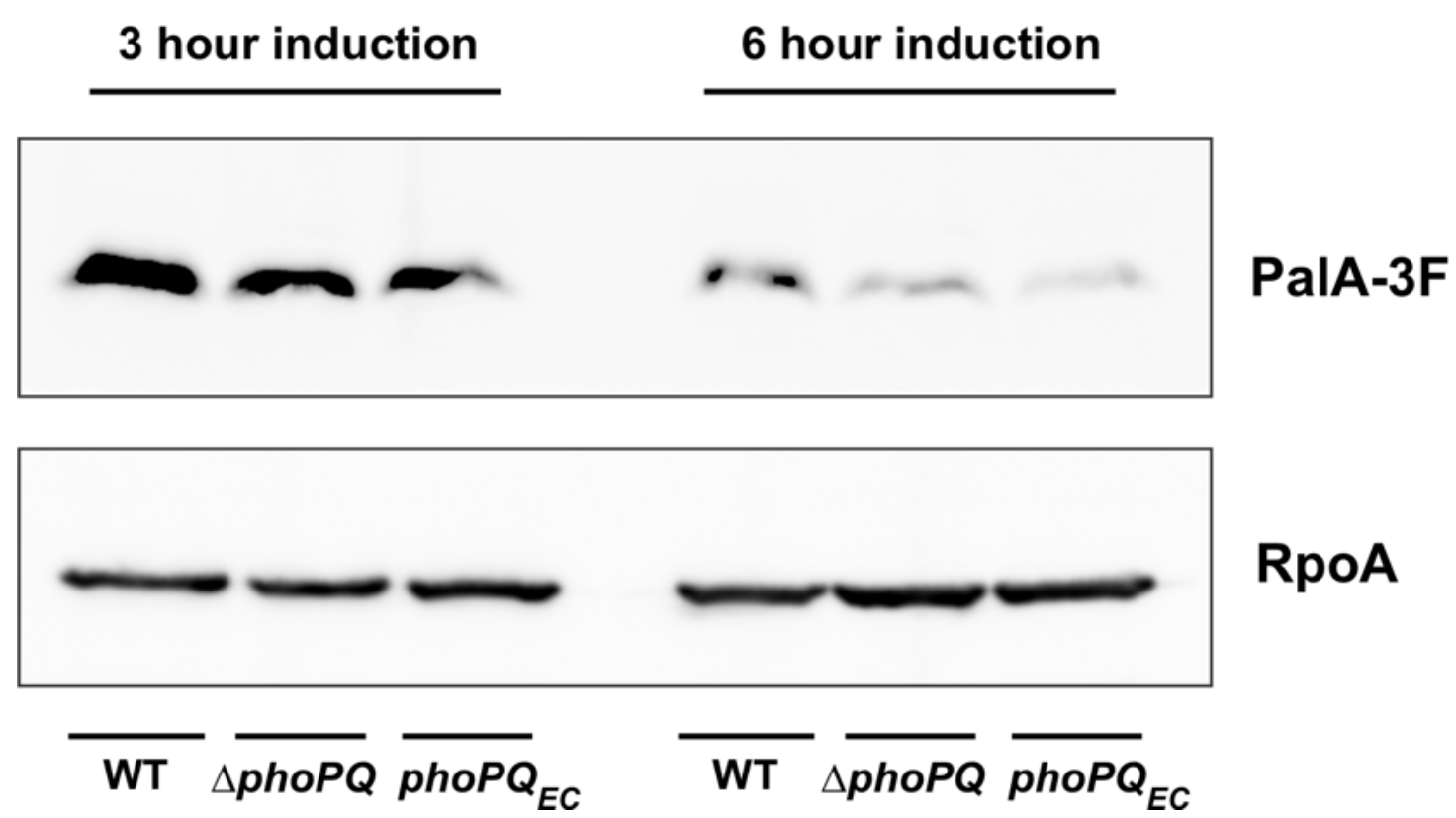

Fig S6: PalA levels are enhanced by native levels PhoQ. WT, Δ*phoPQ*, and *phoPQ*$_{EC}$ strains carrying a low copy plasmid expressing PalA-3F were grown in LB +150 ng/ml aTc. At the indicated time points, whole cell lysates were collected and PalA-3F and RpoA (loading control) were detected by western blot. Results representative of two independent experiments showing similar results.

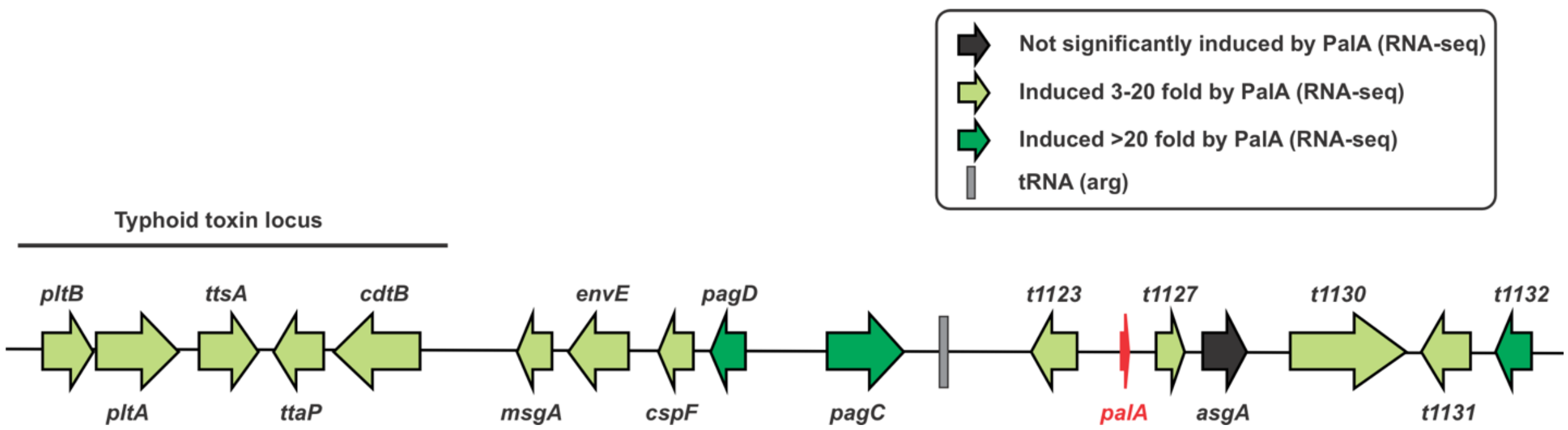

Fig. S7: *palA* is encoded within a cluster of PhoP-induced genes. Gene diagram of the *S*. Typhi *palA* genomic locus highlighting the many PhoP-induced genes in this region of the genome. Fold induction values (colour coded as described in legend) represent that average fold induction values (palA/EV control in the WT strain) from the RNA-seq data presented in the article.
